# Current Applications of Liposomes for the Delivery of Vitamins: A Systematic Review

**DOI:** 10.3390/nano13091557

**Published:** 2023-05-05

**Authors:** Matheus A. Chaves, Letícia S. Ferreira, Lucia Baldino, Samantha C. Pinho, Ernesto Reverchon

**Affiliations:** 1 Laboratory of Encapsulation and Functional Foods (LEnAlis), Department of Food Engineering, School of Animal Science and Food Engineering, University of São Paulo, Av. Duque de Caxias Norte, 225, Pirassununga 13635900, SP, Brazil; matheus.chaves@usp.br (M.A.C.); leticia2.ferreira@usp.br (L.S.F.); samantha@usp.br (S.C.P.); 2 Laboratory of Molecular Morphophysiology and Development (LMMD), Department of Veterinary Medicine, School of Animal Science and Food Engineering, University of São Paulo, Av. Duque de Caxias Norte, 225, Pirassununga 13635900, SP, Brazil; 3 Department of Industrial Engineering, University of Salerno, Via Giovanni Paolo II, 132, 84084 Fisciano, Italy; ereverchon@unisa.it

**Keywords:** vitamins, nanoencapsulation, nanodispersions, phospholipid vesicles, liposomes, cosmetics, food application

## Abstract

Liposomes have been used for several decades for the encapsulation of drugs and bioactives in cosmetics and cosmeceuticals. On the other hand, the use of these phospholipid vesicles in food applications is more recent and is increasing significantly in the last ten years. Although in different stages of technological maturity—in the case of cosmetics, many products are on the market—processes to obtain liposomes suitable for the encapsulation and delivery of bioactives are highly expensive, especially those aiming at scaling up. Among the bioactives proposed for cosmetics and food applications, vitamins are the most frequently used. Despite the differences between the administration routes (oral for food and mainly dermal for cosmetics), some challenges are very similar (e.g., stability, bioactive load, average size, increase in drug bioaccessibility and bioavailability). In the present work, a systematic review of the technological advancements in the nanoencapsulation of vitamins using liposomes and related processes was performed; challenges and future perspectives were also discussed in order to underline the advantages of these drug-loaded biocompatible nanocarriers for cosmetics and food applications.

## 1. Why Encapsulating Vitamins for Food and Cosmetics Application?

Vitamins are organic compounds required for metabolic processes, and not produced by the human body in sufficient amounts. Each vitamin has specific functions in the body and cannot be replaced by any other substance [1]. Having different chemical structures, vitamins can be classified in two main groups: fat-soluble and water-soluble compounds. Thirteen main vitamins have been identified; they are characterized by different ways of action and different beneficial roles in the body. In addition, vitamins play a key role in transforming energy and regulating the metabolism pathways of the human body [2].

**Figure 1:**
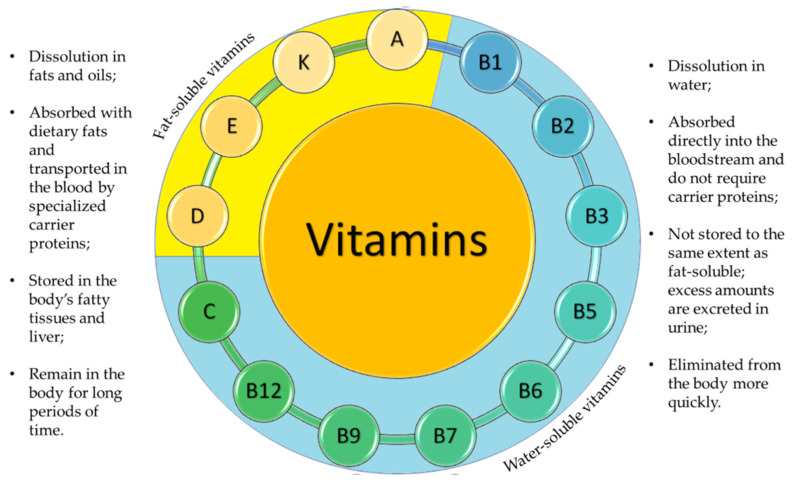
Schematic representation of the main differences between fat-soluble and water-soluble vitamins. Fat-soluble vitamins (A, D, E, and K) are stored in the body’s fatty tissues and liver, whereas water-soluble vitamins (B-complex and C) are not. The differences in solubility, absorption, storage, and excretion can affect the way these vitamins act in the body.

Vitamins A, D, E, and K are compounds that are soluble in fat, meaning that they need a certain amount of fat in the diet to be properly absorbed by the body. In contrast, group B vitamins and vitamin C are soluble in water and cannot be stored in the body, requiring daily intake. Figure 1 provides a schematic representation of the main differences between fat-soluble (hydrophobic) and water-soluble (hydrophilic) vitamins. Vitamins are typically added to food products to fortify them, to replace vitamin losses during processing, or to serve as antioxidants and natural colorants. Table 1 summarizes the chemical structure, sources, and ways of obtainment of the main vitamins. Vitamins are also used in cosmetics for skin care, hair care, and oral health [2]. According to the European Union legislation, vitamins A, E, K, B, and C can all be used in cosmetics as ingredients, except for vitamin D_3_ (cholecalciferol), which is restricted. However, in the USA and Japan, there are no such restrictions [2].

In order to improve their effectiveness, stability, and palatability, vitamins can be encapsulated into delivery carrier systems. This can be especially beneficial for individuals who struggle to obtain adequate amounts of vitamins through their diet or who have specific nutrient deficiencies that require a targeted delivery of these essential nutrients. Encapsulation offers a way to protect vitamins from degradation, to enhance their bioavailability, and to mask any unpleasant taste or odor. This technique refers to the process of enclosing one or more active ingredients, such as vitamins, in a protective carrier, such as liposomes, micelles, or nanoparticles. The encapsulated active ingredients are typically surrounded by a protective layer or membrane that can help shield them from degradation, improve their solubility, target them to specific tissues or cells, and enhance their bioavailability and efficacy. Overall, encapsulation offers several advantages over other delivery methods, such as improved stability, protection from external factors, controlled release, and targeted delivery, making it a promising approach for the development of more effective and efficient delivery systems for vitamins and other bioactive compounds.

### 1.1. Fat-Soluble Vitamins

Vitamin A can be found as retinoids (retinol, retinal, and retinoic acid) and provitamin A carotenoids (mainly β-carotene) [3]. It is important to emphasize that vitamin A is not produced endogenously. The retinol is the first precursor of two vital active metabolites: retinal, which plays an important role in the development of vision, and retinoic acid which acts as an intracellular signal that alters the transcription of a range of genes. Vitamin A is not found directly in plants; however, plants can contain carotenoids, such as β-carotene, which is transformed in vitamin A in the intestine and other body tissues. Therefore, the vitamin A supply is necessarily obtained through ingestion (dietary sources or vitamin supplements) [4]. Foods such as fish, meat (mainly liver), eggs, and whole milk are animal sources of retinol [2,3]. Fruits and vegetables such as carrots, spinach, and mango are sources of carotenoids. Vitamin A is crucial for the growth and development of children and for maintaining the immune system’s function [5]. In adults, a deficiency in vitamin A can negatively impact the immune system, reproductive function, and eyesight, resulting in conditions such as night blindness [3,4,6]. However, overexposure to vitamin A can lead to harmful health effects, including teratogenicity [7]. In the cosmetic industry, vitamin A is widely used due to its ability to delay photoaging effects. Being the main bioactive for skin treatment, it promotes the regeneration of the skin aged by UV radiation, reduces wrinkles, and improves skin elasticity [2]. Vitamin A is not degraded by heat, but it is easily oxidized, and care is required during its processing. To reduce this undesired effect, antioxidants are added to vitamin A products such as, for example, vitamin E [2].

Group D vitamins are composed of ergocalciferol (vitamin D_2_) and cholecalciferol (vitamin D_3_). Vitamin D_3_, or cholecalciferol, is a fat-soluble compound synthesized by the human epidermis by the irradiation of UV light on 7-dehydrocholesterol [8]. The precursor molecule of vitamin D is ergosterol (or 7-dehydrocholesterol), a rigid structure that is inserted by the body when absorbed by the lipid layer of the plasmatic membrane. The production of provitamin D occurs after solar incidence on the aromatic ring of ergosterol. The structure, then, becomes less rigid, promoting an increase in its permeability and, thus, allowing the incorporation of numerous ions into its interior, including calcium. It can be synthesized by the human epidermis or consumed in the form of supplements or fortified foods. A few foods naturally contain vitamin D in their composition: fish, such as salmon and sardines, butter, and eggs being the main sources. Consuming vitamin D_3_ can provide several benefits, including improving calcium absorption in the intestine and maintaining proper levels of calcium in the bloodstream, which helps prevent conditions such as osteoporosis [9]. Unfortunately, a significant portion of the urban population today is deficient in vitamin D_3_, primarily due to a lack of sun exposure and the loss of 7-hydrocholesterol reserves in the epidermis as a result of aging [10]. Vitamin D deficiency can lead to bone diseases and other health problems, such as cancer, asthma, arthritis, hypertension, osteoporosis, and cardiovascular issues. Symptoms of a deficiency may include bone pain and muscle weakness [11]. On the other hand, possible adverse effects regarding the excessive amounts of vitamin D can cause calcium buildup in the blood, leading to nausea, vomiting, and muscle weakness. Long-term overdoses can result in kidney damage [8]. Vitamin D is used in the cosmetic industry as it prevents photo-damage, wrinkles, and other morphological skin changes [2]. Some studies indicated its topical application for the treatment of psoriasis [12]. However, it has an adverse effect on calcium metabolism and limits its use for topical applications [13]. 

Vitamin E is a term used to describe several compounds, including tocopherols and tocotrienols, which are differentiated by the prefixes α, β, γ, and δ. Among them, α-tocopherol is the most common and has a higher bioavailability than other forms of vitamin E [14]. Vegetable oils, such as peanut, soy, palm, corn, safflower, sunflower, and wheat germ are the most important dietary sources of vitamin E. This vitamin plays an essential role in several physiological functions, including acting as an antioxidant, regulating immunity, and providing anti-inflammatory and neuroprotective benefits [15]. Moreover, vitamin E helps protect body tissues from oxidation caused by metabolic processes and external agents while also assisting in the synthesis of vitamin A [2]. High doses of vitamin E, however, can lead to bleeding, particularly in individuals taking blood-thinning medications [14]. However, its incorporation in foods can be challenging, as it is extremely sensitive to high temperatures, light, oxygen, and alkaline conditions, and has low solubility in water [15]. Encapsulation techniques can help to overcome these obstacles allowing the application of vitamin E in foods, cosmetics, and nutraceuticals. In the cosmetic industry, vitamin E is generally used as an antioxidant for the skin, aiding in softening and promoting hydration [2].

Vitamin K can exist in three forms: vitamin K_1_ (phylloquinone, phytonadione, phytomenadione), vitamin K_2_ (menaquinone), or vitamin K_3_ (menadione). Vitamin K_1_ is commonly found in plants, K_2_ is synthesized by bacteria in the human and animal intestines, and K_3_ is a synthetic compound that is converted to K_2_ in the intestinal tract [2,16,17]. Green leafy vegetables, such as spinach, kale, broccoli, and cauliflower, are excellent sources of this vitamin, which is also found in smaller amounts in liver, lean meat, cow’s milk, egg yolks, and whole wheat products [2]. Although structurally different, both vitamins K_1_ and K_2_ can act as cofactors for the enzyme gamma-glutamylcarboxylase, with hepatic and extrahepatic activity. Additionally, vitamin K_2_ plays a vital role in regulating osteoporosis, atherosclerosis, cancer, and inflammatory diseases, with no risk of negative side effects or overdosing [16,17]. Additionally, vitamin K is effective in treating dark circles and bruises on the face, and its application to reduce the effects of bruising after certain dermatological procedures has also been studied [18]. Careful attention must be paid to excessive amounts of vitamin K, as it can interfere with the effectiveness of blood-thinning medications and increase the risk of blood clots [16].

### 1.2. Water-Soluble Vitamins

Group B vitamins are thiamine (B_1_), riboflavin (B_2_), niacin (B_3_), pantothenic acid (B_5_), pyridoxine (B_6_), biotin (B_8_), folacin (B_9_), and cobalamin (B_12_). Vitamin B_1_ can be found in small amounts in brewer’s dried yeast, pork, lamb, beef, poultry, whole grains, nuts, vegetables, and legumes. It is important for carbohydrate breakdown, nerve and muscle function, and healthy skin [2]. Vitamin B_2_ can be sourced from various food items such as milk, dairy products, meat, eggs, and leafy green vegetables. It plays a crucial role in releasing energy from food and promoting the development of healthy skin, vision quality, and growth. Vitamin B_3_ is found in yeast, liver, poultry, lean meats, nuts, and legumes, and is used for the treatment of lipid disorders and cardiovascular diseases, which are essential for growth and hormone synthesis [19]. Pantothenic acid is necessary for the release of energy from food for the production of antibodies and healthy growth, and is present in almost every type of food, and particularly abundant in yeast and animal organs. Vitamin B_12_ is essential for DNA synthesis and red blood cell production, and its deficiency can result in anemia, cognitive impairment, and neurological abnormalities. Vitamins B_5_ and B_12_ are commonly used in skin and hair care products due to their moisturizing, anti-inflammatory, and wound healing properties [20]. Main potential risks regarding the overconsumption of vitamin B may lead to skin flushing, itching (vitamin B_3_), numbness, and tingling sensations (vitamin B_6_), and nerve damage and anemia (vitamin B_9_) [19].

Vitamin C, also known as ascorbic acid, is a commonly used ingredient in both cosmetic and pharmaceutical products due to its powerful antioxidant properties. However, incorporating vitamin C into products poses a significant challenge as its stability must be maintained and delivery to the desired site improved [21]. Vitamin C is naturally occurring in a variety of fruits and vegetables, such as citrus fruits, currants, peppers, parsley, cauliflower, potatoes, sweet potatoes, broccoli, Brussels sprouts, strawberries, guava, and mangoes. In addition to its use as an antioxidant to prevent food and beverage spoilage, vitamin C is essential for the production of collagen, connective tissue, and protein fibers, which provide strength to teeth, gums, muscles, blood vessels, and skin. It also plays an important role in the immune system by aiding white blood cells in fighting infections and facilitating iron absorption within the body [2,21]. Overconsumption of vitamin C can lead to diarrhea, nausea, and abdominal cramps. Long-term excess consumption can lead to kidney stones [21].

## 2. Why Choose Liposomes for the Encapsulation of Vitamins?

In 1965, it was reported, for the first time, that phospholipid molecules were able to instantaneously form closed bilayer vesicles in aqueous media due to the amphiphilic nature of phospholipids [22]. Liposomes are vesicular structures formed by one or more phospholipid bilayers that encapsulate part of the aqueous medium in which they are dispersed [23]. Their average diameters range from 20 nm to several microns. Phospholipids are the main constituents of vesicles, being an amphiphilic molecule in which the hydrophilic polar head groups are oriented towards the aqueous phase and the hydrophobic non-polar hydrocarbon tails are oriented towards each other in an ordered bilayer structure. A wide variety of phospholipids can be used for the production of liposomes, such as eggs, soy, and milk, which are natural and safe sources. The most used phospholipid for the production of liposomes in the food and cosmetic industry is phosphatidylcholine (PC). In addition to PC, phospholipids such as lysophosphatidylcholine (LPC), phosphatidylinositol (PI), phosphatidylethanolamine (PE), and phosphatidylglycerol (PG) can also be used.

When introduced into water, phospholipids tend to group together to form lipid bilayers due to their insolubility in water. By providing the system with sufficient energy through external methods, such as sonication, heating, or homogenization, the negative interaction between the fatty acid molecules and water can be eliminated, allowing the bilayers to organize themselves in a favorable manner. As a result of this process, liposomes can effectively encapsulate hydrophilic compounds in their aqueous core, as well as hydrophobic compounds in the internal regions of the lipid bilayer [24].

While being formed, liposomes acquire various sizes and structural characteristics, such as the number of bilayers [25,26,27]. Size and number of bilayers determine the classification of liposomes in two main types: unilamellar vesicles and multilamellar vesicles, as shown in Table 2. Small unilamellar vesicles (SUVs) have a size ranging from 20 to 200 nm and only contain one bilayer membrane. Large unilamellar vesicles (LUVs) are larger than 200 nm and have a single bilayer membrane. Giant unilamellar vesicles (GUVs) have a size exceeding 1 µm. Multilamellar vesicles (MLVs) contain several concentrically arranged vesicles with a size between 0.5 to 5 µm, whereas multivesicular vesicles (MVVs) have smaller vesicles inside a larger vesicle [12,24]. The size characteristics of liposomes are determined by the production method and the type(s) of phospholipid(s) utilized. SUVs have a higher surface area to volume ratio compared to MLVs. This can result in faster release kinetics and better cellular uptake due to their smaller size and increased surface area. However, they are generally less stable than MLVs, particularly under harsh environmental conditions, which can result in aggregation and fusion with other liposomes. On the other hand, MLVs have a higher loading capacity due to their multiple lamellar layers. In terms of in vitro performance, the choice between SUVs and MLVs will depend on the specific application and the desired characteristics of the liposomes. For example, if rapid drug release and cellular uptake is important, SUVs may be preferred. If long-term stability and high loading capacity are required, MLVs may be preferred [23].

In general, liposomes are one of the most commonly commercialized lipid carriers used for nutraceutical purposes. They are also increasingly studied for their potential to be incorporated into foods for functional applications [28,29]. In the vitamin field, other lipid systems have also been used for encapsulation purposes, such as emulsions, micro/nanoemulsions and solid lipid particles [24]. In this context, some advantages can be pointed out regarding the use of liposomes over these other systems: (i) biocompatibility: the phospholipids used for liposomes production are similar to the phospholipids found in cell membranes and are, thus, less likely to cause adverse reactions; (ii) versatility: liposomes can encapsulate all types of vitamins, hydrophilic, lipophilic, or even both in the same structure due to the amphiphilicity of their structure; (iii) targeted delivery: liposomes can be modified with targeting ligands, such as antibodies or peptides, to enhance their specificity for a particular cell type or tissue; (iv) controlled release: these vesicles can be engineered to release their contents at a specific time or location, improving the therapeutic effect of the vitamins and reducing the need for frequent dosing; and (v) ease of preparation: liposomes can be easily prepared using simple techniques, which makes them a cost-effective drug delivery system compared to others previously mentioned [24,28].

However, despite their versatility, liposomes are physicochemically unstable. This is due to the fact that the lipids present in their structure can undergo natural degradation through oxidation or hydrolysis, or even because the particles can form agglomerates. Liposomes originally show repulsive forces between their particles that provide a certain physical stability, but external factors, such as high temperatures or pH changes, can affect their structure and change the permeability of the bilayer, causing the release of the encapsulated compound or the formation of agglomerates. In order to reduce these undesired effects, a possible solution is the coating of liposomes with biopolymers that can increase their physical stability through steric and electrostatic factors, thus creating a hybrid system. Among the biopolymers used for the coating of liposomes, starches, gelatin, proteins, cellulose, pectin, and chitosan can be mentioned. Other possibilities to increase their overall stability include the use of lyophilization, the incorporation of hydrogenated phospholipids in the lipid bilayers, and the use of cross-linking agents [24,28].

## 3. Methods for the Production of Vitamin-Loaded Liposomes

Generally, a successful encapsulation of bioactive molecules in liposomes, with desired and specific size and structure, depends on several factors, such as the correct choice of the main lipid, the affinity between the liposome and the bioactive of interest, and, most of all, the production method. The latter is considered to be extremely important when encapsulating vitamins for several reasons that may include: (i) the efficiency of encapsulation, as some methods may result in higher encapsulation efficiencies than others; (ii) the stability of the vitamin, as certain methods may be less harmful to them, which is particularly important for sensitive vitamins that can be easily degraded by heat, light, or exposure to oxygen; (iii) the control of particle size and distribution, which are directly related to the release rate of the vitamin from the encapsulating material and also to its bioaccessibility and bioavailability; and (iv) the process scalability, to ensure both consistent quality and efficient production.

**Figure 2:**
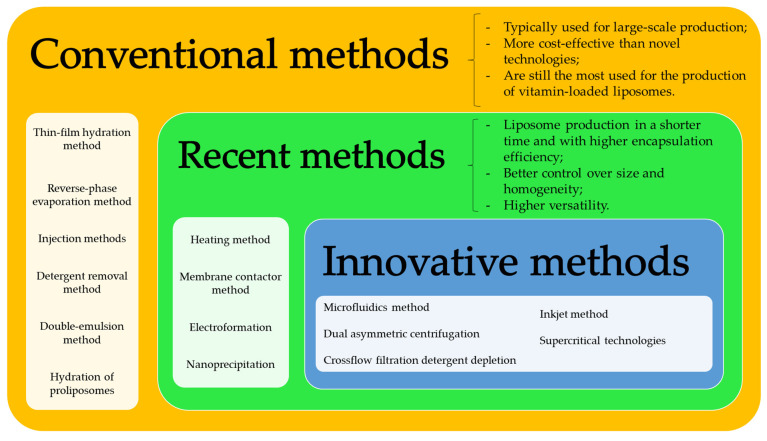
Overview of the conventional and novel methods for liposome production covered in this review. Advantages of each group (conventional vs. recent/innovative) are summarized within the braces.

Conventional methods of liposome production for vitamin encapsulation are still in the spotlight for many researchers due to their simplicity, recognition, and wide range of applications. On the other hand, novel methods have arisen due to their advantages over the conventional ones, such as higher encapsulation efficiency, reduced toxicity, versatility, and scalability. Overall, the production method used for encapsulating vitamins plays a critical role in ensuring the stability, efficacy, and quality of the final product. Figure 2 presents all the methods that will be discussed in this review, whereas Table 3 summarizes the advantages and disadvantages of each one.

### 3.1. Conventional Methods

In the conventional methods, five main steps are basically followed: (i) lipids are dissolved in an organic solvent (ethanol, ether, chloroform, dichloromethane); (ii) the organic solvent is removed using evaporation, rotary evaporation, or distillation; (iii) the resultant lipid layer is hydrated using an aqueous medium (distilled water, buffer solutions, serum-containing media, or physiological saline solutions) and agitated; (iv) vesicles are analyzed and eventually treated by downsizing steps (see Supplementary Material for a detailed description of these methods), depending on the liposome final use; and (v) post-formation processing (purification or sterilization) is carried out to increase the stability of the liposomes [30]. Vitamins must be dissolved in the liquid medium in which they are easily solubilized, i.e., in the organic solvent if lipophilic (A, D, E, and K) or in the hydration medium if hydrophilic (complex B vitamins and C) vitamins are selected.

#### 3.1.1. Thin-Film Hydration (TFH) Method (Bangham Method)

The thin-film hydration technique, also referred as the Bangham method due to the British biochemist that first described it in 1965, is the simplest method used for the preparation of MLVs. It is mostly used for the encapsulation of lipophilic molecules and is based on the production of a thin lipid film by the evaporation at 45–60 °C of an organic solvent from a lipid solvent solution during flask rotation under vacuum and its further hydration using an aqueous media. When small volumes of liposomes are desired, the organic solvent can be dried by using argon steam or dry nitrogen in a fume hood [31]. Hydration processes should be performed above the phase transition temperature of the lipids (e.g., 60–70 °C) for a duration of at least 1–2 h [30]. In addition to the ability to encapsulate both hydrophilic and hydrophobic molecules, the TFH method presents some limitations, such as low encapsulation efficiencies and batch-to-batch variability. The production of liposomes using TFH method is usually followed by a downsizing process to obtain SUVs.

Bi et al. [32] used egg PC, cholesterol (Chol), and vitamin D_3_ (3:1:1 *w*/*w*) to produce vitamin D_3_-loaded MLVs by the TFH method followed by high-pressure homogenization. Obtained liposomes presented a particle size of 169 nm and an encapsulation efficiency (EE%) of 62%. Vitamin B_12_-loaded liposomes were produced by Andrade et al. [33] by the TFH technique followed by ultrasonication, using 1,2-distearoyl-sn-glycero-3-phosphocholine (DSPC), Chol, polyethylene glycol 2000 (PEG200), and amine 1,2-distearoyl-sn-glycero-3-phosphoethanolamine-N-[amino(polyethylene glycol)-2000] (DSPE-PEG2000) in a 52:45:3:0.06 molar ratio. LUVs showed sizes of 116 nm and an EE% of 14%. The authors stated that the low EE% was due to the high hydrophilicity of vitamin B_12_. Campani et al. [34] produced a liposome-based formulation loaded with vitamin K_1_ by the TFH technique followed by extrusion through polycarbonate membranes with decreasing porosity (400, 200, and 100 nm). Lipid films were prepared using soy phosphatidylcholine (SPC) and were rehydrated by PBS at pH 7.4 or aqueous solution at 0.01% *w*/*v* of benzalkonium chloride. Samples produced in these conditions were all in the nanometric range (131–147 nm) and presented vitamin K_1_ encapsulation ratios of 3.4–154.0 µg VK1/mg SPC. Vitamin E-loaded nanoliposomes were efficiently prepared by Qu et al. [35] by using egg lecithin, cholesterol, sodium deoxycholate, and vitamin E, in a 5.8:1:1.1:1.8 mass ratio, by the TFH method followed by sonication and extrusion. The obtained vesicles presented an average hydrodynamic diameter of 231 nm, EE% of 97%, a narrow size distribution (polydispersity index, PDI = 0.217) and high zeta potential (−52.4 mV).

#### 3.1.2. Reverse-Phase Evaporation (RPE) Method

This technique was first described by Szoka and Papahadjopoulos in 1978 [36] and is generally used to encapsulate large amounts of hydrophilic bioactives with high encapsulation efficiencies [37]. Similar to the TFH method, lipids are first dissolved in an organic solvent, such as diethyl ether, chloroform, isopropyl ether, or a mixture of two solvents, in order to form inverted micelles [38]. After the addition of an amount of aqueous phase, water-in-oil (W/O) microemulsions are formed due to the rearrangement of lipids at the interface between water and oil. During this step, a large amount of the aqueous phase is encapsulated within the microemulsion, as well as the hydrophilic molecules. The organic solvent can be slowly removed using a rotary evaporator under vacuum until the conversion of the micelles to a semi-solid viscous gel-like structure is obtained. The gradual removal of the solvent favors the disruption of the gel and promotes the formation of LUVs [30]. As discussed for the TFH method, a downstream step, such as sonication or extrusion, is required to reduce the average size of liposomes and to obtain a narrow size distribution [39]. The main drawbacks related to this technique include: (i) the use of a large amount of organic solvents and the presence of residual solvent at the end of the process; (ii) the high complexity and difficulty to industrial scaling up; (iii) the unsuitability to be employed for the encapsulation of sensitive molecules due to the long-lasting contact with the organic solvent; (iv) the time-consuming process; and (v) the sterile boundary is quite hard to establish [40]. 

There are a few studies exploring this method for the encapsulation of vitamins, mainly due to their high degradability rates. Favarin et al. [41] employed the RPE method to encapsulate vitamin C into liposomes. In their study, the aqueous phase was composed of polysorbate 80, at pH 3.65, and vitamin C, whereas the organic phase was composed of phospholipid Lipoid^®^ S100, cholesterol, and ethanol. The inverted micelles were formed after the addition of the aqueous phase to the organic phase under ultrasonic conditions. The mixture was, then, submitted to a slow evaporation in a rotary evaporator operating at 80 rpm and 35 °C for the removal of the organic solvent, and the formation of the gel-like structure. Liposomes were produced after the addition of the aqueous phase under agitation. To homogenize the vesicles size, samples were extruded using membranes with pores of 0.45 μm and 0.22 μm. Vitamin C-loaded liposomes showed a mean size of 160 nm and PDI of 0.23, besides a slight negative zeta potential of −7.3 mV and an EE% equal to 19%.

#### 3.1.3. Injection Methods

The injection methods are based on the dissolution of lipids into an organic solvent (ethanol or diethyl ether) and the further injection of the resulting solution into an aqueous phase. Among all the liposome production techniques, the injection methods are suitable to operate continuously [42].

- Ethanol injection (EI) method: this method was first described by Batzri and Korn [43] and is based on the dissolution of phospholipids in ethanol and, then, the injection of the solution into a rapidly stirred aqueous phase [43]. Part of the ethanol evaporates upon contact with the aqueous phase, producing a lipid film that forms liposomes upon hydration. Vesicles are formed due to the immediate diffusion of the ethanol in the aqueous medium, leading the lipid molecules to precipitate and to form bilayered planar structures that tend to liposomes [44]. A change in the solubility of lipids leads to the spontaneous formation of vesicles that encapsulate a small volume of the aqueous phase. This method is relatively simple and produces liposomes characterized by a high entrapment efficiency of hydrophobic molecules. SUVs can be formed if proper process parameters, such as low lipid concentrations and fast rate of injection, are used. A disadvantage of EI is that the encapsulation efficiency of hydrophilic compounds is low, and the control of both size and size distribution of the resulting liposomes can be challenging. On the other hand, some advantages of this method include: (i) it is a straightforward method, which makes it a popular choice for liposome production; (ii) its high reproducibility; and (iii) its easy scale up, making it a practical choice for commercial production [44]. However, this method is limited by the need for subsequent processing to solvent evaporation and the residual content [45]. The use of the EI method to produce liposomes for food or cosmetics is usually hindered by the low encapsulation efficiency of hydrophilic bioactive molecules such as vitamin C. This occurs because hydrophilic bioactives tend to be preferentially retained in the external aqueous phase of vesicles instead of in their small aqueous core [46]. Charcosset et al. [47] developed a continuous process for the ethanol injection method coupled with membrane extrusion, in which vitamin E-loaded MLVs were produced using Lipoid^®^ S100 as the source of phospholipids. Liposomes were stored in a vacuumed double jacketed reactor in which the ethanol residue was constantly removed. Using this method, volumes between 60 mL to 3 L of liposomes with sizes ranging from 89 to 118 nm were produced;

- Ether injection method: this method requires the dissolution of lipids in diethyl ether and, then, a slow injection of the solution into an aqueous phase under high pressure. The ether rapidly evaporates upon contact with the aqueous phase under warming, resulting in a lipid film that forms liposomes upon hydration [48]. This method results in a concentrated liposomal (LUVs) product with a narrow size distribution and high entrapment efficiency [45]. Some differences can be pointed out between this method and EI: (i) ethanol is a polar solvent, unlike ether that is non-polar; this can affect the lipid solubility and the self-assembly of lipids in liposomes, resulting in differences in size, size distribution, and other properties of liposomes; (ii) ether injection method may be more complex and time-consuming than EI method; and (iii) different effects on the stability of lipids and liposomes as the use of ether may cause a larger lipid degradation or oxidation than the use of ethanol due to the formation of peroxides [30]. However, an advantage of using the ether injection method consists of the more efficient removal of the organic solvent from the final product [30]. For food purposes, it is generally preferable to use ethanol instead of ether. Ether is highly flammable and can pose a fire hazard, which may be a concern in a food production setting. Ethanol, on the other hand, is a safer solvent that is commonly used in food processing and is generally recognized as safe (GRAS) by the US Food and Drug Administration (FDA). Ethanol is also a polar solvent, thus, more compatible with the polar environment of aqueous foods. The ether injection method is not commonly used for the encapsulation of vitamins in liposomes, basically because of its high toxicity [49].

#### 3.1.4. Detergent Removal (Depletion) Method

The detergent depletion method is a mild process capable of producing highly homogeneous liposomes. This method is based on the formation of mixed micelles of detergents and lipids and the further removal of the detergent to form LUVs. The size of vesicles is based on the rate at which the detergent is removed from the formulation and the initial detergent to phospholipid ratio [50]. Detergents, such as sodium cholate, sodium deoxycholate, and octyl glycoside, are often used in the initial stages of liposome preparation to solubilize the lipids and to form a homogeneous solution. However, detergents can also destabilize the liposome membrane and lead to aggregation and fusion of the liposomes. To remove the detergent and to obtain stable liposomes, various methods are used. One of the most commonly used methods is dialysis [51]. Using this method, the detergent containing the liposome dispersion is submitted to a dialysis step. This process is typically carried out over several hours to several days, depending on the amount of detergent and the desired degree of detergent removal [52]. Another method for detergent removal is the use of adsorbents such as Bio-Beads [53]. The obtained liposomes are, then, separated from the beads containing adsorbed detergent molecules by filtration or centrifugation, resulting in detergent-free liposomes [53].

Vitamin-loaded liposomes can also be produced by the detergent depletion method. In this case, vitamins should be solubilized with the lipids in a detergent-containing solution. Hydrophilic compounds are generally encapsulated more efficiently in liposomes produced by detergent removal than hydrophobic bioactives since the latter may be more prone to being removed along with the detergent during the depletion process. However, in the case of food applications, the choice of detergents used in the liposome production should be carefully considered; some safety concerns or regulatory restrictions may apply. Secondly, this process can be time-consuming and labor-intensive, and may not be easily scalable to commercial production levels. Some detergents, such as Tween 20, Tween 80, and Brij-35, are considered safe for food applications, as they are approved by FDA and European Food Safety Authority (EFSA). Triton X-100 cannot longer be used for food applications, but its use in cosmetics is still feasible due to the regulation approved by several agencies. It is important to notice that the choice of the detergent can affect the properties of the resulting liposomes, including their size, stability, and encapsulation efficiency [45]. Therefore, it is necessary to carefully optimize the detergent concentration and preparation method to achieve the desired properties of the liposomes for the intended final application. Additionally, it is important to ensure that any residual detergent is removed from the liposomes during the detergent depletion process to avoid potential health risks [54]. When producing liposomes for cosmetic applications, it is important to choose a detergent that is safe for use on the skin and does not cause irritation or other adverse effects on humans.

#### 3.1.5. Double Emulsion Method

The double emulsion method, also known as the water-in-oil-in-water (W/O/W) emulsion method, is a technique for encapsulating both hydrophilic and hydrophobic molecules in liposomes [55]. Hydrophilic bioactives, such as proteins, are more suitable to be encapsulated using this technique due to the multiple aqueous phases involved in the emulsification process. In this context, the method involves two emulsions, one inside the other one [56]. The first step is to prepare a W/O emulsion. This is performed by mixing a small amount of an aqueous solution containing the hydrophilic compound of interest with an immiscible organic solvent, such as chloroform or toluene, and a lipid solution. The mixture is vigorously shaken to create small droplets of the aqueous solution surrounded by a lipid bilayer. The second step is to create a second emulsion by adding the W/O emulsion to a large amount of the aqueous phase containing a stabilizer, such as a surfactant or a polymer. This is typically carried out by sonication or homogenization. The resulting W/O/W emulsion contains small droplets of the aqueous phase plus the hydrophilic compound, surrounded by a lipid bilayer, which in turn is surrounded by a second aqueous phase. Liposomes are formed after the removal of the organic solvent by stripping gas or vacuum pressure, which leads to the direct contact between the external and internal oil–water phases and the formation of the lipid bilayer [57]. The double emulsion method presents some advantages over the other liposome production methods, such as high encapsulation efficiency of hydrophilic compounds, drug-controlled release, and versatility. However, this process can be time-consuming since it requires several steps to form liposomes, besides the low yield, and the need for specialized equipment, such as sonicators or homogenizers [39].

A modification of this method, named “freeze-drying of double emulsions”, has been efficiently used to produce liposomes characterized by improved stability and prolonged shelf life. This technique involves three main steps: (i) the formation of a double emulsion by the emulsification of an aqueous phase with a lipid phase, followed by a new emulsification with an external aqueous phase; (ii) the freezing of the double emulsion to produce a frozen matrix of liposomes, which helps to stabilize the liposomes and to prevent aggregation or coalescing phenomena; and (iii) the freeze-drying process, in which the frozen matrix is submitted to freeze drying for the removal of water through sublimation under vacuum. This step transforms the frozen matrix of liposomes into a dry powder that can be easily reconstituted with water or other appropriate solvents. Some advantages related to this method include: (i) improved stability, making them suitable for long-term storage and transportation; (ii) preservation of structural integrity, thus, maintaining their biological activity; and (iii) flexibility, as liposomes can be produced at different sizes and compositions depending on the final destination. However, this technique shows some drawbacks, such as the requirement for specialized equipment, the potential for loss of encapsulated compounds during the freeze-drying process, and some damage to the freeze-dryer apparatus due to residual solvents [58].

Li et al. [59] produced complex liposomes containing medium-chain fatty acids (MCFAs) and vitamin C using the double emulsion method followed by dynamic high pressure microfluidization. A mass ratio of 100:25:4 soybean phospholipids, cholesterol, and vitamin E, altogether with an ethanolic solution containing MCFAs, was used throughout the production. The procedure was followed by the injection of a small volume of twice-distilled water under vigorous stirring at 50 °C. The primary emulsion was formed after the evaporation of part of the solvent under reduced pressure. The aqueous phase composed of twice-distilled water, Tween 80, and vitamin C was, then, incorporated into the organic phase. After agitation, the residual solvent was removed through rotary evaporation under reduced pressure. These authors obtained liposomes with a mean diameter of approximately 93 nm, encapsulation efficiencies of MCFAs and vitamin C of 49% and 64%, respectively, and a good stability over 90 days at 4 °C. In another study, Yang et al. [60] used the same protocol as Li et al. [59] to produce complex liposomes encapsulating MCFAs and vitamin C, but these authors added a freeze-drying step at the end of the process to increase vesicle stability. The reconstituted liposomes presented a mean diameter of 110 nm and an encapsulation efficiency of MCFAs and vitamin C of 44% and 62%, respectively. Vesicles remained stable for 60 days at 4 °C. In a more recent study, Pattnaik and Mishra [61] produced a multivitamin (A, D, B_9_, and B_12_) liposome using the double emulsion technique and a mix of soy lecithin and vegetable oil blend, in addition to a polymer solution containing milk protein isolate and trehalose at different concentrations. Liposomes were stable, with size varying from 143 to 396 nm and zeta potential from −20 to −33.5 mV. Interestingly, these authors observed that hydrophilic vitamins showed lower entrapment efficiency than the hydrophobic vitamins (vitamin B_9_, EE% = 78–97.6% and vitamin B_12_, EE% = 96–99.9% vs. vitamin A, EE% > 99.9% and vitamin D, EE% > 98%) and stated that this behavior was probably due to the interaction between the lipophilic vitamins with the hydrophobic tails of phospholipids during freeze-drying, which might have caused a protective effect over the vitamins. 

#### 3.1.6. Hydration of Proliposomes

This method of production uses a powder mixture of dry phospholipids and bioactives, called proliposomes. Several techniques can be used to obtain these powders, including fluidized bed, spray drying, freeze-drying, coating of micronized sugars, milling, and supercritical techniques. Proliposomes are defined as dry, free-flowing powders that contain the bioactives to be encapsulated [62]. Upon hydration under appropriate conditions, MLVs are formed. Their solid form confers stability and offers advantages, such as improved transport convenience, storage, distribution, and dosage. Proliposomes can be manufactured using industrialized procedures as tablets or capsules, which eliminates the stability problems of liquid liposomes and may increase the oral bioavailability of bioactives [63].

Spray drying is a commonly used technique in the pharmaceutical and food industries for the production of powdered materials. This method is widely used for the encapsulation of oils, flavors, and fragrances. Its large application is mostly due to its ability to evaporate moisture rapidly from a sample, maintaining a low temperature in the particles [64]. Wall materials, such as polysaccharides and proteins, are commonly used throughout the process [65]. In the liposome field, spray drying acts as a post-processing step to convert the liquid form of dispersions to a high stable solid form [66]. First, the liposome dispersion must be produced, selecting the desired bioactive molecule by any method of interest. This solution is, then, atomized in small droplets using a spray nozzle and dried using a hot gas stream. The resulting powder is collected and can be used for further processing or packaging. Spray drying has several advantages for the production of bioactive-loaded liposomes. It is a fast and efficient method for producing large quantities of powdered material, and it can be easily scaled up for commercial production [67]. The resulting powder is also stable and can be stored for long periods of time without degradation. However, there are some challenges associated with spray drying regarding the encapsulation of vitamins. This process can cause damage to the liposome structure, leading to a loss of the encapsulated material. Additionally, the high temperatures used during spray drying can degrade sensitive vitamins [68]. However, some recent studies on thermosensitive vitamins, such as vitamin B_1_, B_9_, and vitamin B_12_, showed that they may not experience significant degradation during the spray drying process, depending on the wall material and process parameters [69,70]. The microencapsulation of lipophilic vitamins such as vitamin D using spray drying is rare due to the mandatory water-dispersed form that is needed for the processing. In addition, the porosity of the resultant material can act as an advertisement about the risk of degradation of more sensitive materials due to oxygen exposure [68]. The use of lower temperatures or shorter drying times during the spray drying process may prevent early thermal degradation.

Spray drying was used to produce β-carotene-loaded proliposomes by Toniazzo et al. [71]. Phospholipids, sucrose, and β-carotene were solubilized in anhydrous ethanol and treated by spray drying using an inlet temperature of 90 °C and outlet temperature of 85 °C, at a flow rate of 10 mL/min in a 1 mm diameter spray nozzle with co-current airflow. Proliposomes were, then, rehydrated with deionized water and thickeners xanthan and guar gums were used as stabilizers. The MLVs produced by this method were useful to protect β-carotene over 95 days of storage with an EE% up to 96% when a 0.10% *w*/*w* mixture of xanthan and guar guns was incorporated during the hydration step.

### 3.2. Recent and Innovative Methods

Novel methods for liposome production have been developed and improved over the years, with the aim of overcoming the drawbacks related to the traditional methods. Differently from the liposomes produced using the conventional methods, vesicles produced by these new methods are mainly unilamellar and show a more homogeneous size distribution (lower values of PDI) [72]. Therefore, post-processing techniques, such as sonication or extrusion, are rarely required. Moreover, conventional methods are based on the use of organic solvents and detergents, which can limit their use in foods and cosmetics, and are often not environmentally friendly. Currently, research efforts around liposomes focused on the optimization of green technologies that can be scaled up to industrial levels.

#### 3.2.1. Heating Method

This method was first described by Mozafari et al. [73] and involves the hydration of phospholipids with a 3% *v*/*v* glycerol solution at increasing temperature up to 60 °C or 120 °C. The processing temperature depends on the absence or presence of Chol. The use of glycerol is due to its physicochemical characteristics, such as water solubility and non-toxicity, besides its ability to avoid sedimentation or coagulation of vesicles. Sterilization of the resulting liposomes is not required due to the high temperature already used during processing (120 °C), leading to cost reduction. The heating method has been improved over the years resulting in a new method called the Mozafari method, in which large-scale production of liposomes can be achieved without the need for the prehydration step of raw materials and without the use of toxic solvents or detergents [74]. Although vitamins are mostly thermosensitive, equally sensible molecules such as DNA are being incubated at room temperature with preformed liposomes produced by the heating method [73].

#### 3.2.2. Membrane Contactor Method

In this technique, a porous membrane with a defined pore size is used to separate two fluid compartments [75,76]. The organic phase (generally ethanol + lipids) is flowed across one side of the membrane, while an aqueous solution is flowed across the other side. The two fluids come in contact at the pores of the membrane, and liposomes are formed thanks to the presence of water. Liposomes then diffuse tangentially through the pores of the membrane and are collected on the surface of the other size. The organic solvent is removed by evaporation under reduced pressure. This method offers some advantages over traditional liposome production methods. Firstly, it is a continuous process, able to be scaled up, thus allowing for the production of large quantities of liposomes. Secondly, it is a gentle method, with minimal shear stress, which reduces the potential damage to liposomes or to the encapsulated molecules. Thirdly, it offers better control over the size and size distribution of the liposomes, as the pore size of the membrane can be precisely controlled [77]. Additionally, membranes can be regenerated by washing using a water/ethanol mixture.

This method was already used to produce vitamin E-loaded liposomes [78]. Vesicles were produced using 20–50 mg/mL of 1-palmitoyl-2-oleoyl-sn-glycero-3-phosphocholine (POPC) or Lipoid^®^ E80, 5–12.5 mg/mL of a stabilizer (Chol, stearic acid or cocoa butter), and up to 5 mg/mL of vitamin E. Shear stress on the membrane surface ranged from 0.80 to 16 Pa. Mean particle size under optimal conditions was 84 and 59 nm for Lipoid^®^ E80 and POPC liposomes, respectively. EE% up to 99% was obtained in MLVs produced using Lipoid^®^ E80.

#### 3.2.3. Electroformation

This method was developed by Angelova and Dimitrov [79] and consists of the formation of GUVs under electric fields by the hydration of a lipid film deposited on electrodes. This method relies on the ability of lipids to self-assemble in bilayer structures in the presence of an electric field. The electric field creates a potential difference across the lipid solution, causing the lipid molecules to move towards the electrode, where they self-assemble in bilayer structures. The electroformation process typically involves the following steps: (i) preparation of the lipid solution, typically by mixing lipids in a suitable organic solvent; (ii) deposition of the lipid solution on an electrode or a conductive substrate, typically made of glass, quartz, or indium tin oxide; (iii) application of an electric field to the lipid solution by using two electrodes, typically made of platinum or gold; and (iv) collection of the vesicles from the electrode surface and further purification using size exclusion chromatography, ultracentrifugation, or dialysis [80]. The main disadvantages of this method include its low production yield and limited scalability.

Vitamin E-loaded GUVs were prepared by Di Pasquale et al. [81] in which the influence of vitamin E on the membrane organization was corroborated by SANS and fluorescence microscopy. Samples were produced using 1,2-dipalmitoyl-sn-glycero-3-phosphocholine (DPPC), 1,2-dioleoyl-sn-glycero-3-phosphocholine (DOPC), and Chol at a 37.5:37.5:25 mole ratio and vitamin E at a mole fraction of 0.10.

#### 3.2.4. Nanoprecipitation

This method for liposome production can be easily scaled up and allows for the creation of liposomes in a single step without the need to homogenize the vesicles to achieve a uniform size. The procedure involves the mixing of a lipid solution with an aqueous solution, and the lipid molecules come together spontaneously to create liposomes since they are less soluble in the aqueous medium. By altering the composition and processing parameters, the size of the liposomes can be controlled. Due to the self-assembly mechanism, the resulting liposomes are characterized by a narrow size range and can be manufactured with a high degree of consistency [82].

Jash and Rizvi [83] utilized the nanoprecipitation method to produce coated liposomes loaded with both vitamin C and vitamin E. They employed polyethylene glycol and acetic acid sodium acetate buffer with a pH of 4.5 as solvent and non-solvent, respectively. The surface of the liposomes was coated with a polyanionic block copolymer Eudragit^®^ S100, and the resulting solution was slowly added dropwise to the acetic acid-sodium acetate buffer. The coated liposomes were then concentrated using centrifugation. The study’s primary findings revealed that Eudragit^®^ S100 safeguarded the encapsulated cargo from the harsh gastric environment and enabled targeted pH-triggered release in simulated intestinal conditions.

#### 3.2.5. Microfluidics Method

Microfluidics refers to a collection of techniques that use narrow channels with micrometric cross-sectional dimensions (ranging from 5 to 500 µm) to manipulate fluid flows. It offers several advantages, such as axial mixing regulated by diffusion and continuous operation at low volumes. Microfluidic techniques have been proven to produce uniformly dispersed liposomes and allow for precise control over liposome size by adjusting either the volumetric flow rate or the total flow rate [84]. The process operates at low Reynolds numbers (laminar flow) and requires diffusive mass transfer. A detailed description of liposome formation via microfluidics can be found in Carugo et al. [85]. In brief, the process involves pushing a stream of lipids dissolved in alcohol through the central channel of a microfluidic device, where it is sheathed and crossed by two lateral streams of a water phase. By adjusting the volumetric flow rate ratio and the total flow rate, the size of the focused stream can be adjusted. Liposome formation occurs when the lipids solubilized in alcohol diffuse into the water, and the water diffuses into the alcohol until the alcohol concentration decreases below the lipid solubility limit, resulting in self-assembly of lipids to form liposomes [85]. However, there are some limitations to the microfluidic approach, including the use of organic solvents, the requirement for delicate mechanical agitation, and the challenges associated with scaling-up production.

Dalmoro et al. [86] prepared vitamin D_3_ and vitamin K_2_-loaded uncoated and chitosan-coated nanoliposomes by a novel simil-microfluidic device. PC, Chol, and each vitamin were diluted with alcohol, whereas deionized water was used as the hydration solution. Higher encapsulation efficiencies were obtained for vitamin D_3_-loaded liposomes (EE% = 88–98%) and vitamin K_2_-loaded liposomes (EE% = 95–98%) at chitosan concentrations of 0.01% and 0.005% *w*/*v*, respectively.

#### 3.2.6. Dual Asymmetric Centrifugation (DAC)

The DAC method employs a distinctive kind of centrifugation where a vial holding the mixture of liposomes is spun around its own center and the main axis. While the primary rotation forces the sample material outward, the rotation of the vial around its own center moves the sample material inward as it adheres to the vial. This inward movement is effective when the material is viscous enough and can adhere to the vial material. The resulting vesicular phospholipid gel can then be diluted to produce liposomes. These gels are particularly useful for obtaining a high content of liposomes suitable for use in products such as creams, lotions, or hydrogels [87]. The DAC method has also been shown to produce a highly uniform population of liposomes with improved drug release properties compared to other methods [88]. Furthermore, the equipment is of a small size, easy to operate, and offers good reproducibility. Liposomes with high EE% for water-soluble bioactives can be produced by this method, which does not require the use of organic solvents. Drawbacks of this method include the high shear force and the need for formulations that contain a high amount of phospholipids to increase the viscosity and to form the vesicular gel [89]. To the best of our knowledge, there is no literature on the encapsulation of vitamins by the DAC method; however, it appears as a suitable method to encapsulate mainly water-soluble vitamins.

#### 3.2.7. Cross-Flow Filtration Detergent Depletion

Peschka et al. [90] developed a method that combines the conventional detergent depletion technique with a cross-flow filtration system to provide a fast solution for detergent removal. This system comprises a starting reservoir, a pump, a filtration device with a membrane system, tubing with an integrated rotary slide valve, and a manometer to monitor the retentate pressure. Increasing the pressure on the membrane leads to the rapid removal of the detergent. The mixed micelle solution in the starting reservoir undergoes tangential filtration through a single membrane or membrane cassettes with a selected molecular weight cutoff. This cross-flow filtration process enables the production of liposomes with uniform size, homogeneity, and high stability [89]. Compared with other detergent removal methods, large quantities of liposomes can be produced in a significantly shorter time [91]. This method also allows for the production of sterile products by using sterile filtered mixed micelles and autoclaved devices. Additionally, the wasted filtrate can be recycled to minimize production costs [91].

#### 3.2.8. Inkjet Method

The ethanol injection method has been modernized by the development of the inkjet method, which allows for precise control over liposome size and has the potential for scalable production [92]. Amphiphilic compounds are dissolved in ethanol and printed into an aqueous solution using an inkjet device, resulting in uniform liposome droplets in the range of 20–100 nm. Additionally, the inkjet method has been utilized to produce unilamellar lipid vesicles by transforming a bioactive solution into a jet of uniform droplets, which then collide with a solution containing a lipid bilayer membrane at the liquid/air interface. This sequence of events leads to the formation of each lipid vesicle, as the membrane undergoes deformation, collapse, and separation [93]. Although it is not currently used for the encapsulation of vitamins in liposomes, inkjets have been used to produce edible films for oral delivery of B-complex vitamins [94].

#### 3.2.9. Supercritical Technologies

Supercritical fluid technologies have emerged as attractive methods for producing liposomes [56]. CO_2_ is the most commonly used gas in supercritical fluid technology for several reasons, including low cost, non-toxicity, safety, and large availability. It is therefore considered a safe gas for use in food, as well as pharmaceutical and cosmetic applications [56]. Most processes of liposome production that use supercritical CO_2_ (sc-CO_2_) involve dissolving a lipid mixture in a solution of CO_2_ at high pressure and temperature. The CO_2_ solution then acts as a solvent for the lipids, leading them to self-assemble and form vesicles. The pressure and temperature of the CO_2_ solution can be easily controlled to modify the size and shape of the samples. One of the key advantages of sc-CO_2_-based processes is the absence of organic solvents, which can be toxic and difficult to remove from the final product. In addition to being non-toxic, CO_2_ readily evaporates, leaving no residue in the final product. Over the years, many supercritical technologies for liposomes production have been explored, and some of them are discussed below.

- Depressurization of expanded liquid organic solution into aqueous solution (DELOS-susp): this is a compressed fluid-based method that enables the reproducible and scalable production of nanovesicular systems with exceptional physicochemical properties, including uniformity, morphology, and particle size [95]. To prepare the samples, the reagents are initially dissolved in an organic solvent and subsequently treated using pressurized CO_2_ until saturation. Then, the sample is rapidly depressurized from the bottom, experiencing a significant pressure drop from 10 MPa to ambient pressure. CO_2_ molecules are released from phospholipid bilayers to temporarily disrupt them into highly dispersed phospholipids that undergo a rapid reorganization due to hydrophobic and van der Waals interactions, and then packing themselves into liposomes [96,97]. This process produces small and uniform liposomes due to the high rate of depressurization and can be used to encapsulate thermo-sensitive materials since it works under slight conditions [98]. Adaptations of this method have been proposed to remove the need for organic solvents or surfactants during production, some of them still resulting in SUVs at high storage stability [99];

- Depressurization of expanded solution into aqueous media (DESAM): this is an alternative dense gas technology, in which pressure requirements for liposome production are reduced to 4–5.5 MPa [100]. This method involves dissolving lipids in a solvent and pressurizing the solution with a dense gas to create an expanded lipid solution. The expanded solution is, then, released in a controlled manner into heated aqueous media, with pressure maintained through the addition of more dense gas. Care is taken to keep the pressurization and expansion below a certain threshold to avoid solute precipitation. The dense gas and solvent can be separated and reused. The resulting fine droplets disperse the lipid in the aqueous phase and improve component interaction, resulting in uniform liposome formation. The high temperature in the vesicle formation chamber can also aid in removing organic solvent from the product [100]. Liposomes produced by this method are mainly unilamellar, with sizes ranging from 50 to 200 nm, PDI not exceeding 0.29, and is highly stable for periods of 8 months [100]. Recently, a continuous process named nano-carrier by a continuous dense gas (NADEG) technique appeared as an evolution of the DESAM method [101,102];

- Rapid expansion of supercritical solution (RESS): this is a technology currently used for micronization, co-precipitation, and encapsulation. Lipids are dissolved in a mixture of sc-CO_2_ plus 5–10% *v*/*v* of ethanol within an extractor. This primary dissolution in a co-solvent (ethanol) is strictly necessary because the natural phospholipids are poorly soluble in sc-CO_2_ [103]. This solution is then released through a heated small nozzle in a low-pressure chamber and mixed with an aqueous solution. A rapid depressurization follows, and the pressure drop results in the lipids desolvation, which favors the formation of layers around the droplets due to solute supersaturation. Small particles are obtained from the gas stream [104,105]. This process produces small particles with a uniform size. However, this method shows problems such as the difficult separation between vesicles and co-solvents during depressurization, which increases production costs [106]. Nevertheless, the RESS method is one of the most studied supercritical technologies for vitamin-loaded liposome production [107,108,109,110,111,112]. Han et al. [112] produced vitamin E acetate-loaded liposomes using an optimized RESS process without any organic solvent. Operating conditions were controlled using the single-factor analysis and the response surface methodology combined with Box–Behnken design. Samples were produced using polyvinyl acetate grafted phospholipids and vitamin E acetate in a 6.35:1 mass ratio and resulted in vesicles characterized by EE% = 93%, size of 247 nm, PDI of 0.295, and zeta potential of −42.5 mV. Jiao et al. [108] also used the Box–Behnken design to optimize the process parameters for the production of vitamin C-loaded liposomes using PC as wall material. Vesicles presented size of 270 nm, PDI of 0.254, zeta potential of −41.7 mV, and EE% of 75%. Sharifi et al. [111] produced ironized multivitamin-loaded liposomes containing lecithin, Chol, iron sulfate, and hydrophilic and hydrophobic vitamins (C and E, respectively) by a new venturi-based method called Vent-RESS, in which RESS was combined with Bernoulli principles. Liposomes with unimodal size distribution were obtained and EE% of bioactive molecules were improved when operating pressure increased from 12 to 18 MPa. The Vent-RESS process was also used by Jash, Ubeyitogullari, and Rizvi [110] to produce vitamin C–vitamin E co-loaded liposomes in milk fat globule membrane phospholipids (MFGM) or sunflower phosphatidylcholine (SFPC). These authors verified that MFGM-based ULVs were smaller in size than SFPC-based ones (533 nm vs. 761 nm, respectively), with higher zeta potential (−57 mV vs. −37 mV, respectively);

- Supercritical reverse phase evaporation process (scRPE): this is a batch method developed by Otake et al. [113] that enables the efficient formation of liposomes using a one-step process. It acts similarly to the RPE method but, in this case, sc-CO_2_ substitutes the organic solvent. It involves the mixing of sc-CO_2_, lipids, and ethanol and then, the introduction of small amounts of water to generate a liposome dispersion through an emulsion formation. The procedure is carried out in a stirred volume cell at a temperature above the lipid phase transition temperature. As the aqueous solution is gradually added to the reactor, sc-CO_2_ is released, resulting in the formation of liposomes upon depressurization [97,114]. Some years later, Otake et al. [115] optimized the scRPE method in a way that organic solvents were no longer needed. Zhao and Temelli [116] developed a similar process in which liposomes are formed by simple pressurization and depressurization of a sc-CO_2_ lipid-based aqueous solution. Liposomes size can vary from 100 nm to 1.2 µm by using this method, being SUVs or MLVs [117];

- Supercritical antisolvent (SAS): it involves a continuous spraying of an organic solution of phospholipids into sc-CO_2_, which serves as an antisolvent for phospholipid precipitation. As soon as sc-CO_2_ contacts the liquid phospholipid phase, it quickly diffuses and divides the liquid phase in tiny droplets. At the same time, the organic solvent evaporates from the droplets as a consequence of the dissolution in sc-CO_2_. This mass transfer creates a supersaturation of phospholipids within the droplets, leading to the formation of small phospholipid particles through nucleation and aggregation. A pure CO_2_ washing step can be performed to remove any trace of the organic solvent. Finally, spherical micro- or nanoliposomes are formed upon hydration with an aqueous buffer. Xia et al. [118] produced vitamin D_3_-loaded proliposomes using a SAS based technology. Hydrogenated PC was used as a lipid source. Conditions such as T = 45 °C, P = 8 MPa, and 15% *w*/*w* lipid to vitamin D_3_ ratio resulted in hydrated liposomes with an EE% = 100% and an effective loading of 12.9%;

- Supercritical assisted liposome formation (SuperSomes): this is a continuous sc-CO_2_-based process proposed by Reverchon and co-workers [119] in which, differently from the other methods, water particles are first formed by atomization and then, covered by lipids dissolved in an expanded liquid mixture. The expanded liquid mixture is composed of phospholipids, ethanol, and sc-CO_2_. The main idea is that lipids reorganize themselves around the water droplets forming inverted micelles, which tend to form liposomes as soon as they come in contact with a water pool located at the bottom of the vessel [120,121]. Process parameters, as water flow rate, injector diameter, phospholipid concentration, pressure, and gas to liquid ratio, have been constantly optimized during SuperSomes studies [122]. This apparatus has been efficiently used to encapsulate both hydrophobic and hydrophilic bioactive molecules [123,124,125,126]. The process is reproducible, therefore, allowing for a good control of vesicle size distribution, and nanometric vesicles at high EE%. Recent studies showed the feasibility of using SuperSomes apparatus to encapsulate vitamin D_3_ into nanoliposomes [127,128]. In both studies, liposomes were produced using different ratios of hydrogenated soy and nonhydrogenated egg yolk phospholipids. In Chaves et al. [127], samples produced using only egg yolk phosphatidylcholine presented sizes of 132 nm. Furthermore, a 10 mL/min water flow rate also led to highly homogeneous vesicles produced using a maximum of 20% of hydrogenated soy phospholipids with a size of 218 nm, PDI of 0.253, and an EE% of 89%. In Chaves et al. [128], the effect of the incorporation of vitamin D_3_ in curcumin-loaded liposomes was investigated. The addition of vitamin D_3_ reduced the overall size of liposomes from approximately 220 nm to 130 nm, but also promoted a decrease in EE% of curcumin. The authors stated that this behavior was probably due to the competition between the two hydrophobic bioactives for the inner region of lipid bilayers;

- Aerosol solvent extraction system (ASES): the ASES method, initially intended for the creation of a sterile product composed of a biodegradable carrier and a molecule embedded within it, involves the spraying of organic liquids through a nozzle into a bulk of sc-CO_2_ [129]. This facilitates the rapid precipitation of solutes from the solution, which can be easily dried with circulating sc-CO_2_, allowing for the simple removal of residual solvent from the precipitates. This technique has been applied to liposome preparation, resulting in the production of dry and reconstitutable pharmaceutical liposomes that are suitable for large-scale manufacturing [130,131];

- Particles from gas saturated solution (PGSS): this is a cutting-edge method that uses supercritical fluids to produce particles of a precise size. This process operates at mild temperatures, generally between 40–60 °C, in an inert environment, and uses CO_2_ and water as solvents [132]. The PGSS-drying method provides an alternative to conventional techniques such as spray drying and freeze drying. One of its significant benefits is the efficient atomization achieved through rapid gas release and expansion during depressurization from supercritical to ambient conditions. Additionally, this method allows drying at lower temperatures in the spray tower, minimizing the exposure of the bioactive material to high temperatures that may cause damage [133]. Another advantage of this technique is its ability to process carrier materials with low melting temperatures, which conventional spray drying cannot handle.

In summary, the use of traditional methods for creating and reducing the size of liposomes is still popular due to their ease of implementation and lack of requirement for advanced equipment. Although some of these methods can be effective for processing certain vitamins, they may also cause structural changes that could impact their functionality. Additionally, these methods are not always easily scalable for an industrial production. In recent years, there is a growing need for innovative manufacturing techniques in nanotechnology that can encapsulate both hydrophilic and hydrophobic molecules without the use of organic solvents or complex equipment, which is of interest considering the need for multivitamin products. Additionally, the challenge of maintaining system stability for liposomes loaded with hydrophilic materials requires new strategies to achieve optimal loading while targeting delivery to the intended site. The lack of scalability in some conventional liposome formation processes and low encapsulation efficiencies are still major obstacles to mass production. To address these challenges, it is essential to explore novel formation methods and to integrate new technologies to produce advanced liposome formulations that are not only suitable for industrial-scale production, but also highly effective for clinical applications. Therefore, much work still needs to be performed in terms of scaling, designing, controlling, and optimizing liposome formation processes [30].

## 4. How to Measure the Effectiveness of Liposomes in Encapsulating Vitamins?

In 2018, the Center for Drug Evaluation and Research of Food and Drug Administration (CDER-FDA, Silver Spring, MD, USA) developed a guidance summarizing the main characterization techniques that producers should consider when developing liposomes at an industrial scale. In this context, the liposome formulation should be able to contain and retain as much as possible the molecule in the appropriate structure. Vesicles should be characterized in terms of morphology, surface characteristics, encapsulation efficiency, bioactive loading, particle size, phase transition temperature, in vitro release of the molecule, leakage rate from liposomes throughout shelf life, and integrity changes in response to changes in factors, such as salt concentration, pH, temperature, or the addition of other excipients.

Liposome composition, production method, and bilayer membrane rigidity can all impact EE%. EE% is calculated as the percentage of bioactives inside the liposomes compared to the total amount of bioactive used. Generally, an encapsulation efficiency of over 50% is considered good. Separation of the free bioactive is necessary to quantify the amount within liposomes. Several techniques are used for separation, including chromatography, gravitation or centrifugation, dialysis membrane, and ultracentrifugation. Indirect and direct methods are used to determine EE%. Conventional techniques for measuring drug concentration include UV–Vis and fluorescence spectroscopy, enzyme or protein-based assays, HPLC, UPLC, LC-MS, and GC-MS. ESR and ^1^H NMR can also be used for bioactive quantification. Table 4 summarizes some studies in which EE% tests were performed on vitamin-loaded liposomes. Solvents and other chemicals commonly used to disrupt the liposome membranes, in addition to the techniques carried out to quantify the amount of hydrophobic and/or hydrophilic vitamins released from vesicles, are also presented.

Table 4 shows that different authors have obtained different encapsulation efficiencies when using liposomes to encapsulate the same vitamin. Although the methods for vitamin quantification are normally well-established, sometimes the method used to produce the liposomes is not the most suitable for a particular vitamin. In this case, the polarity of the molecule plays a crucial role in the choice. Fan et al. [137] attributed the low encapsulation of vitamin A in liposomes to its reactive polyolefin structure, but found that increasing the loading content of vitamin A improved EE%. Conversely, they found that an increase in loading rate decreased the EE% of vitamin E due to a resultant higher viscosity of the vesicles. Pezeshky et al. [136] attributed the low EE% of vitamin A palmitate in nanoliposomes produced by the TFH method to the low hydrophilicity and the size of its structure. The authors also noted that vitamin A palmitate is sensitive to light, oxygen, and organic solvents, making degradation during the process more likely. Cansell, Moussaoui, and Lefrançois [138] observed low entrapment of vitamin B_1_ in liposomes using the TFH method, attributing this behavior to the kind of vesicles formed during the process (MLVs), which usually present low EE% at low lipid concentrations. Xanthan gum was found to help retain vitamin B_1_ by coating the outer surface of the liposomes and thus prisoning a higher content of the vitamin through hydrogen bounding and/or electrostatic interactions. Farhang et al. [149] attributed low values of EE% for liposomes containing water-soluble ascorbic acid to the formation of MLVs when using milk phospholipids and a dehydration/rehydration method. Andrade et al. [33] found that the ethanol injection method was more efficient in producing vitamin B_12_-loaded liposomes with higher encapsulation efficiency than the TFH method, also due to the hydrophilicity of this vitamin. Sharifi et al. [111] found that increasing pressure resulted in increased EE% of vitamin C and vitamin E in loaded liposomes using a supercritical method, which was attributed to a higher solubility of coating materials in sc-CO_2_ at higher pressures. Additionally, higher EE% of vitamin C was observed when using higher amounts of aqueous cargo throughout the process.

## 5. Applications of Vitamin-Loaded Liposomes

### 5.1. In Foods

Liposomes have become increasingly popular in the food industry for functional purposes due to their ability to increase bioactive dissolution rate and bioavailability, protect sensitive ingredients, improve stability during processing, storage and digestion, and to confine undesirable flavors. They have also been used to achieve controlled release to specific targets. The encapsulation of food bioactives using liposomes has been investigated, and there is a wide range of opportunities for research in real applications of liposomes in different food formulations. A proper application of bioactive-loaded liposomes in the food industry should involve excipient materials and bioactives that are generally recognized as safe, fully incorporated within the liposomal structure, and not reactive with the core ingredients. Liposomes can be used to encapsulate an aqueous phase in order to decrease the vapor pressure of a matrix, allowing for the lowering of the water activity without decreasing the moisture content, and thus, preventing the growth of microorganisms in foods that contain nutrients such as proteins or sugars [161]. Studies on the evaluation of shelf-life and sensorial acceptance, as well as oral processing, digestibility, and bioaccessibility of encapsulated compounds in liposomes designed for food applications, should receive more attention in the literature.

Vitamins are not easily incorporated into foods. Liposoluble vitamins are hardly introduced into aqueous-based food formulations and are easily oxidized in the air. Most vitamins are also thermolabile and can be readily degraded after thermal treatments such as pasteurization. On the other hand, the addition of vitamins into liposomes can protect their activity, in addition to increasing their absorption and bioavailability [86]. Some studies have been carried out in order to test the effects of the incorporation of vitamin-loaded liposomes in food matrices. Marsanasco et al. [150] produced SPC-based liposomes encapsulating vitamins E and C for orange juice fortification. The hydration of a thin-film lipid method was used to prepare these vesicles. To substitute cholesterol, which is related to health issues as atherosclerosis and high blood pressure, the authors tested other molecules to increase the rigidity of membranes such as stearic acid (SA) and calcium stearate (CaS). The lipid films were constituted of mixtures of SPC and SA or CaS in a 1:0.25 molar ratio and vitamin E from a stock solution, and were further hydrated with acetic acid 3% *v*/*v* containing vitamin C to form liposome dispersions. These dispersions were, then, added to orange juice samples, which were pasteurized at 65 °C for 30 min and stored at 4 °C. In another study, Marsanasco et al. [162] incorporated SPC:SA- and SPC:CaS-based vitamin E-folic acid co-loaded liposomes to enrich chocolate milk. Vesicles were prepared using the thin lipid film hydration at a 1:0.25 molar ratio of SPC and SA or CaS. Vitamin E was incorporated in the lipids before the hydration step, whereas folic acid was incorporated in the hydration buffer. Samples were pasteurized and then, added in a 1/100 ratio in chocolate milk kept at 4 °C. These authors observed a protective action of vitamin E over folic acid as oxidative stability of folic acid remained unchanged in the presence of vitamin E after pasteurization. 

Banville, Vuillemard, and Lacroix [152] produced vitamin D-loaded liposomes aiming to fortify cheddar cheese. Vesicles were prepared by hydrating a proliposome mixture (Pro-Lipo-Duo^TM^, Lucas Meyer, Chelles, France) using a vitamin D solution. The resulting MLVs were then incorporated into raw milk, which was employed for the cheese manufacturing. The results showed that vitamin D-loaded liposomes promoted a higher final concentration of the vitamin in the processed cheeses than a commercial water-soluble vitamin D emulsion (Vitex-D). Wechtersbach, Ulrih, and Cigić [163] incorporated vitamin C-loaded liposomes based in DPPC and Chol in both apple juice and fermented milk that underwent pasteurization at 72 °C; they observed higher retentions of vitamins after encapsulation than when added in free form. These authors also verified a higher retention of the molecule in vesicles containing DPPC and Chol than those containing DPPC alone. Mohammadi, Ghanbarzadeh, and Hamishehkar [164] produced vitamin D_3_-loaded liposomes aiming at beverage fortification. Samples were produced using SPC and Chol at various concentrations by the thin-film hydration method. The resulting MLVs were subjected to homogenization at 60 °C for 15 min and to probe sonication at 20 kHz and 70% of strength in order to decrease their size and turn them undetectable by the human eye. Size of vesicles were up to approximately 87 nm after 30 days of refrigerated storage for samples produced at a 50:10 PC:Chol ratio with an EE% up to 95%. Vitamin D_3_-loaded liposomes were also incorporated into dark and white chocolate by Didar [165,166]. The ethanol injection method was used to prepare these liposomes based on phospholipids and Chol. A concentration of 5 µg/10 g of vitamin D_3_ in chocolate was fixed for free form-enriched and encapsulated-enriched samples. The author verified a reduction of vitamin D_3_ after 60 days of storage in samples fortified with the free form of the vitamin instead of encapsulation in liposomes. Moreover, no impacts on rheological, colorimetric or sensory characteristics of chocolate were observed in the samples enriched with liposomes. Another study regarding the enrichment of foods using vitamin D_3_ was conducted by Chaves et al. [167], in which pineapple yogurt was fortified with nanoliposomes co-encapsulating vitamin D_3_ and curcumin. The nanovesicles were produced using different ratios of purified and unpurified phospholipids by the hydration of proliposomes, which in turn were produced by coating of micronize sucrose. Dispersions were produced upon hydration of proliposomes, which were stabilized by xanthan and guar gums at a 0.02% *w*/*v* concentration. Liposomes were incorporated into formulated pineapple yogurts at 5% *v*/*v* and maintained under refrigeration at 4 °C. These authors verified that an increased concentration of purified phospholipids was beneficial to increase the EE% of both bioactives in dispersions. Liu et al. [168] produced vitamin C-loaded nanoliposomes by self-assembly of alginate and chitosan to enrich mandarin juice. Samples were prepared by the thin lipid film hydration method combined with microfluidization at 120 MPa for 2 cycles. The main ingredients included SPC, Chol, Tween 80, and vitamin E at a 6:1:1.8:0.12 ratio. Samples were rehydrated using PBS 0.05 M at pH 7.4 containing 5 mg/mL of vitamin C. Vesicles were, then, coated with alginate and chitosan using electrostatic deposition. Finally, liposomes were incorporated into mandarin juice samples and pasteurized at 90 °C for 10 s and stored at 4 °C. Liposome coverage by alginate and chitosan was responsible for an increase in size and a reduction in zeta potential of the samples, in addition to promoting aggregation of vesicles at day 90. However, lipid peroxidation and vitamin C release were lower than non-coated samples, suggesting a protective effect. It is worth mentioning that several other bioactive molecules that are not vitamins have been incorporated into liposomes and tested in food matrices, such as *Clove* oil in tofu [169], nisin in Minas frescal cheese [170], rutin for chocolate coating [171], quercetin in cornstarch [172], betanin in gummy candy [173], baicalin in mushrooms [174], basil essential oil in pork [175], ginger extract in wheat bread [176], and fish oil in yogurts [177].

It is important to note that the risk of allergenicity due to liposomes depends on the source and purity of the phospholipids used in the liposome preparation. To minimize this undesired effect, liposome preparations can undergo rigorous purification steps to remove potential allergenic components. For example, liposomes can be subjected to multiple purification steps to remove any residual allergenic proteins or other components [26].

### 5.2. In Cosmetics

Nanotechnology-based approaches in cosmetics are growing exponentially with the aim of developing novel formulations that can confer aesthetic and therapeutic benefits to people [178,179]. In particular, such cosmetic formulations are referred to as “cosmeceuticals” when they have both cosmetic and medicinal functions [180]. Liposome-based nanoformulations are gaining particular interest since they can represent a promising strategy to prepare antiperspirants, creams, lipsticks, deodorants, moisturizers, hair care products, etc., and can also be successfully used to deliver vitamins, such as vitamin A, B_12_, E, K, antioxidants (such as coenzyme Q10, lycopene, carotenoids, etc.), and other bioactive molecules [46,143,181].

In the literature, in vitro and in vivo studies demonstrated the improved cosmeceutical effect of bioactives when incorporated into vesicles, e.g., in reducing pigmentation disorders, skin aging, and solar exposure problems. These positive effects are due to the incorporation of the bioactives into vesicles that favor the penetration through the stratum corneum of the skin and promotes their activity at the damaged site [182,183]. For instance, the loading of vitamin C into liposomes, by facilitating its percutaneous transport, can increase its concentration in the dermis more than five times compared with the application of the pure vitamin [184]. The encapsulation of vitamin B_12_ in vesicles and its slow release enhances the bioactive absorption and bioavailability, in addition to the protection of the vitamin from the degradation induced by heat, light, air, and improper storage [143]. Jiao et al. [142], to improve vitamin C (VC) and folic acid (FA) stability, co-loaded these antioxidant molecules in liposomes (VCFA-Lip) and chitosan-coated liposomes (CS-VCFA-Lip). The mean vesicles size of VCFA-Lip and CS-VCFA-Lip was 138 nm and 249 nm, respectively, whereas the encapsulation efficiency of both drugs in CS-VCFA-Lip was much higher than that measured for VCFA-Lip. Moreover, the stability study revealed that the chitosan coating can efficiently improve the physical stability of VCFA-Lip. According to Dreier et al. [185], the primary mechanism of action for liposomes is their rupture and fusion with the lipids in the stratum corneum. However, the efficacy of liposomes in delivering bioactives to the skin is impacted by various factors, including their size, surface charge, number of layers (uni- or multi-lamellar vesicles), and the flexibility of the bilayer. A research study conducted by Touti et al. [186] demonstrated that systems possessing higher lipid bilayer flexibility can pass through narrow skin constrictions more efficiently. In contrast, multi-layered liposomes with high flexibility and larger dimensions can penetrate deeper into the skin than smaller vesicles due to abrasion and the loss of outer layers during skin diffusion. Additionally, ultra-deformable lipid carriers that are less than 150 nm in size can penetrate the skin and release active molecules in the deeper layers without adsorption on keratinocytes. The positive charge of phospholipid carriers can also enhance transdermal transport through the skin by promoting stronger electrostatic interactions with the skin surface, which facilitates liposome accumulation on or within hair follicles, enabling them to diffuse more easily. Choi et al. [187] showed that flexible cationic liposomes can promote a larger penetration as compared to their less charged counterparts.

Currently, the two most popular techniques for synthesizing liposomes for cosmeceuticals are the same used for foods, namely, the TFH method and the ethanol injection method. Both are low-cost, easy to use, and versatile methods that create liposomes with different properties. However, as aforementioned throughout this review, the first method produces liposomes that are heterogeneous in size and shape, thus, requiring further steps such as sonication or extrusion to create small, uniform vesicles, making it difficult to use on a large scale. The second offers advantages such as simplicity, scalability, and the ability to produce small-sized liposomes, but it is limited in its ability to encapsulate hydrophilic antioxidants, such as vitamin C [46]. To overcome these limitations, advanced techniques, such as supercritical and microfluidic technologies, that can control size, encapsulation efficiency, and the structural heterogeneity of liposomes through automatic and programmable systems, have shown promise, being considered particularly effective alternatives for liposome assembly to both food and cosmetical applications [46].

## 6. Discussion and Concluding Remarks

Based on the data and methods presented in this review, it appears that exploring the field of vitamin encapsulation in liposomes is a relevant avenue for both the food and cosmetics industries. Several methods of liposome production, from the most conventional to the highly innovative ones, have been described and the application of liposomes in vitamin incorporation was found and contextualized. However, the scale up and the use of liposomes in food and cosmetic products are in distinct technological stages. It is worth mentioning that while there are already liposome-based cosmetic products available on the market, the same cannot be said for food products.

**Figure 3:**
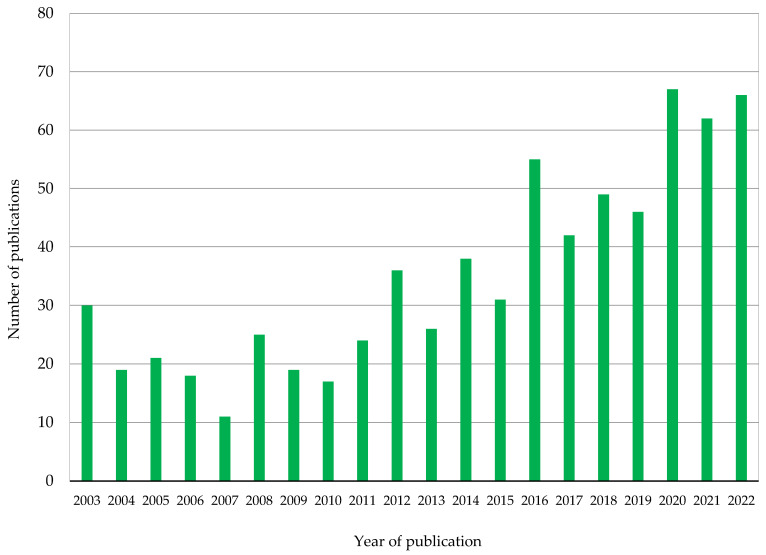
Evolution of the publications about the encapsulation of vitamins in liposomes over the last 20 years.

It is quite important to highlight that the number of publications on this subject has been constantly growing over the last 20 years, but particularly in the last ten years, according to Figure 3.

In the case of foods, the oral route is the main challenge to be faced, mainly due to the known sensitivity of liposomes to the gastric environment. Most vitamins should be released at the end of the gastric phase or only in the intestine to be absorbed, which often requires the liposomes to be gastro resistant. This is one of the main challenges in vitamin-loaded liposomes that needs to be overcome, and some approaches have been tried, such as the coating using polysaccharides (e.g., chitosan and pectin) [188,189]. However, studies about the digestion (both in vitro and in vivo) to determine the bioaccessibility and bioavailability of vitamin-loaded liposomes in food products are still scarce. Other challenges that can be cited for the liposomes to be used in foods are: (i) the high price of phospholipids when considering a large scale; and (ii) restrictions about the use of organic solvents, which is a limitation for the use of some of the most common methods to produce liposomes already scaled up.

On the other hand, as the main route for the application of vitamin-loaded liposomes in cosmetics is through the skin, the elasticity and flexibility of the vesicles are pointed out among the main challenges, together with their average size. Some important rewards come from the topical administration in comparison to the oral route, such as the possibility of lower fluctuations in plasma bioactive levels, site-specific delivery, avoidance of first-pass metabolism, and better patient compliance [190]. Furthermore, as the skin is the first defensive barrier against external factors and prevents several bioactives from penetrating the underlying layers or going into systemic circulation, there are some limitations to deliver the active ingredient to the target site [191]. Ultradeformable liposomes, as transfersomes, ethosomes, niosomes, and transethosomes are the new generation of elastic liposomes suitable for a more effective transdermal delivery of therapeutics, including vitamins [190]. Other challenges related to the use of liposomes in cosmetic products are the low drug loading, physical and chemical instability, and scarce reproducibility of the results [192]. 

Therefore, although both types of utilization (foods and cosmetics) of vitamin-loaded liposomes are in different stages of technological maturity, the development of new technologies to face the challenges presented in this work can be mutually beneficial.

## Figures and Tables

**Table 1:** Chemical structure, ways of obtainment, and main sources of vitamins in foods.

Vitamin	Chemical Structure	Way of Obtainment	Food Sources
A (retinol)	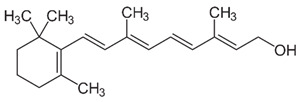	Intake of dietary sources or vitamin supplements	Fish, meat, eggs, whole milk, carrots, spinach, and mango
B_1_ (thiamine)	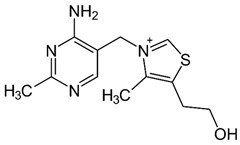	Intake of dietary sources or vitamin supplements	Brewer’s dried yeast, pork, lamb, beef, poultry, whole grains, nuts, vegetables, and legumes
B_2_ (riboflavin)	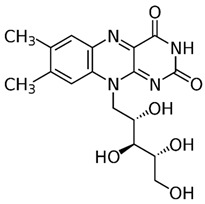	Plants and animal cells	Milk and milk products, meat, eggs, and leafy green vegetables
B_3_ (niacin)	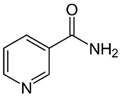	Intake of dietary sources or vitamin supplements	Yeast, liver, poultry, lean meats, nuts, and legumes
B_5_ (pantothenic acid)	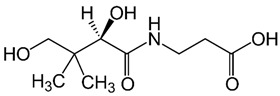	Intake of dietary sources or cosmetic products	Organ of animals, eggs, milk, vegetables, legumes, and whole grain cereals
B_6_ (pyridoxine)	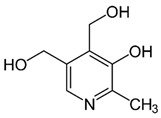	Intake of dietary sources or vitamin supplements	Fruits, vegetables, and grains
B_7_ (biotin)	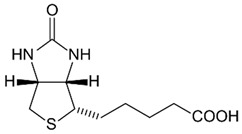	Intake of dietary sources or vitamin supplements	Chicken liver, beef liver, egg yolk, peanuts, sunflower seeds, almonds, salmon, and pork chop
B_9_ (folacin)	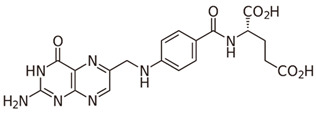	Intake of dietary sources, fortified foods or vitamin supplements	Peanuts, sunflower seeds, lentils, chickpeas, asparagus, spinach, chicken liver, calf liver, cheese, hazelnuts, and avocados
B_12_ (cobalamin)	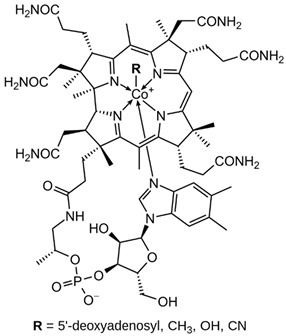	Intake of dietary sources or vitamin supplements	Milk and dairy products, eggs, and salmon
C (ascorbic acid)	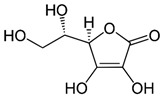	Intake of dietary sources or vitamin supplements	Citric fruits, currants, peppers, parsley, cauliflower, potatoes, sweet potatoes, broccoli, brussels sprouts, strawberries, guava, and mango
D_2_ (ergocalciferol)	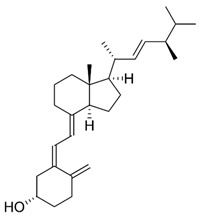	Intake as vitamin D supplement	Mushrooms (portobello, crimini, shitake)
D_3_ (cholecalciferol)	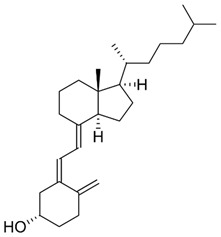	Synthesized by the human epidermis or consumed in the form of supplements or fortified foods	Fish, as salmon and sardines, butter, and eggs
E (α-tocopherol)	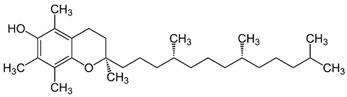	Intake of dietary sources or vitamin supplements	Vegetable oils of peanut, soya, palm, corn, safflower, sunflower, and wheat germ
K_1_ (phylloquinone)	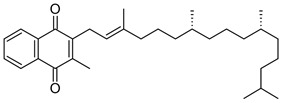	Plants	Green leafy vegetables, liver, lean meat, cow’s milk, egg yolks, and whole wheat products
K_2_ (menaquinone)	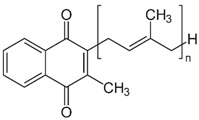	Synthesized by bacteria in the human and animal intestines	-
K_3_ (menadione)	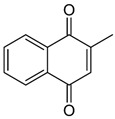	Converted to K_2_ in the intestinal tract	-

**Table 2:** Classification of liposomes based on size and lamellarity.

Type of Liposome	Abbreviation	Characteristic	Diameter	Schematic Representation
Small unilamellar vesicles	SUV	Small unilamellar vesicles	20 to 200 nm	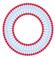
Large unilamellar vesicles	LUV	Unilamellar liposomes with average diameters higher than SUV	Above 200 nm	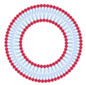
Giant unilamellar vesicles	GUV	Unilamellar liposomes with average diameters higher than LUV	Higher than 1 µm	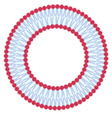
Multilamellar vesicles	MLV	Several concentrically arranged vesicles	0.5 to 5 µm	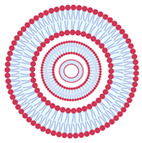
Multivesicular vesicles	MVV	Smaller vesicles within a vesicle of large size	Higher than 1 µm	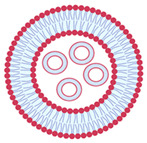

**Table 3:** Advantages and disadvantages related to the methods for liposome production.

Production Methods		Advantages	Disadvantages
Conventional techniques	Thin-film hydration method	✓ Easy to scale up ✓ Straightforward approach ✓ Compatibility with different lipids ✓ Simple method with low cost	✓ Multiple steps ✓ Post-processing is required for size reduction ✓ Water-soluble bioactives may exhibit low EE% ✓ Removal of organic solvents is needed ✓ Sterilization is required
	Reverse-phase evaporation method	✓ Simple and rapid process ✓ High encapsulation efficiency	✓ Time-consuming ✓ Large amounts of organic solvents are needed ✓ Post-processing is required for size reduction ✓ Sterilization is required
	Injection methods	✓ Easy to scale up ✓ Ethanol injection is simple, rapid and reproducible ✓ Ether injection forms a concentrated liposome with high EE%	✓ Removal of solvents is needed ✓ Post-processing is required for size reduction ✓ Macromolecules may inactivate in presence of ethanol
	Detergent removal method	✓ Simple process ✓ Suitable particle size distribution	✓ Low concentration of liposomes ✓ Low EE% for hydrophobic molecules ✓ Time-consuming
	Double emulsion method	✓ Biodegradability ✓ Versatility ✓ Suitable particle size distribution	✓ Multiple steps ✓ Possible leakage of hydrophilic bioactives during production ✓ Post-processing is required for size reduction
	Hydration of proliposomes	✓ Versatility ✓ Easy to scale up ✓ Higher stability when in dried form	✓ Heterogeneity of vesicles ✓ Production variability ✓ Limited loading capacity
Novel techniques	Heating method	✓ Simple and rapid process ✓ No organic solvents ✓ Sterilization is not needed	✓ Multiple steps ✓ Possible degradation of thermosensitive molecules ✓ Post-processing is required for size reduction
	Membrane contactor method	✓ Simple and fast process ✓ Suitable particle size distribution ✓ No organic solvents	✓ Possibility of clogging the pores ✓ High temperature ✓ Membrane/filter fragility ✓ Sterilization is required
	Electroformation	✓ Rapid process ✓ High purity ✓ Adaptable conditions ✓ Formation of GUVs with high EE%	✓ Unknown underlying mechanism ✓ High cost of electrodes ✓ Only suitable for very low ionic strength
	Nanoprecipitation	✓ Simple method ✓ Use of biocompatible solvent	✓ Post-processing is required for size reduction ✓ Sterilization/aseptic processing is required
	Microfluidic method	✓ Simple method ✓ Suitable particle size distribution	✓ Difficulty to remove the organic solvents ✓ Unsuitable for bulk production ✓ High cost of microfluidic channels
	Dual asymmetric centrifugation	✓ Easy to operate ✓ Reproducibility ✓ Suitable particle size distribution ✓ High EE% for water-soluble bioactives ✓ No organic solvents	✓ High amount of phospholipids is required ✓ Batch scale production
	Crossflow filtration detergent depletion	✓ Rapid process ✓ Suitable particle size distribution ✓ Sterilization is not required ✓ Water filtrate can be recycled	✓ Use of detergents ✓ Difficulty to scale up
	Inkjet method	✓ Suitable particle size distribution ✓ High EE%	✓ Specific equipment is needed ✓ Removal of ethanol is required
	Supercritical technologies	✓ Versatility ✓ Small number of unit operations ✓ Low solvent residue ✓ Used for bioactives with low solubility ✓ High efficiency ✓ Environmentally friendly ✓ Suitable particle size distribution ✓ Reproducibility	✓ High pressure ✓ Possible agglomeration of particles at the end of the process ✓ May involve nozzle blockage ✓ May involve high cost for implementation

**Table 4:** Chemicals and methods currently used for the extraction and quantification of vitamins in liposomes.

Vitamin	Liposome Type/Size	Chemical Used to Demulsify/Precipitate the Liposomes	Method for Vitamin Quantification	EE%	Reference
Vitamin A	SUV	Methanol	HPLC	50.6–56.2%	[134]
Vitamin A	MLV	Chloroform/Methanol 2:1 *v*/*v*	UV–Vis spectrophotometry	99%	[135]
Vitamin A	MLV/SUV	Chloroform	HPLC	15.8%	[136]
Vitamins A, D, E, and K	n.s.	Tween 80	HPLC	20–100%	[137]
Vitamin B_1_	MLV	n-octyl β-D-glucopyranoside	Optical density	31.2%	[138]
Vitamin B_1_	n.s.	PBS	HPLC	97%	[139]
Vitamin B_2_	n.s.	Triton X-100	Indirect method (photolysis)	42.3%	[140]
Vitamin B_5_	n.s.	Chloroform/Methanol	Direct method (mass weight)	75%	[141]
Vitamin B_9_	n.s.	Ethanol	Ultrafiltration in centrifuge tube followed by HPLC	87.4%	[142]
Vitamin B_12_	MLV	Methanol/Water 1:10 *v*/*v*	HPLC	70%	[143]
Vitamin B_12_	LUV	-	Centrifugation followed by UV–Vis spectroscopy	27%	[33]
Vitamins B_12_, D_2_, and E	SUV	Ethanol	UV–Vis spectrophotometry	56–76%	[144]
Vitamin C	n.s.	Ethanol	Dialysis followed by UV–Vis spectrophotometry	77.9%	[145]
Vitamin C	n.s.	Methanol, chloroform, and ammonium buffer	HPLC	99.2%	[146]
Vitamin C	n.s.	Ethanol	HPLC	75.4%	[108]
Vitamins C and E	n.s.	Triton X-100/DMSO	UV–Vis spectrophotometry	93–95%	[83]
Vitamins C and E	n.s.	Assay kit BC1235	Indirect method	98.5%	[147]
Vitamin C	SUV/LUV	Meta-phosphoric acid	Titration with indophenol solution	94.2%	[148]
Vitamin C	SUV/MLV	-	Gel permeation chromatography	5–16%	[149]
Vitamins C and E	MLV/MVV	Protamine sulfate solution followed by Triton X-100	Protamine aggregation method followed by UV–Vis spectrophotometry	12–88%	[111]
Vitamins C and E	MLV	Acetic acid and protamine solution	UV–Vis spectrophotometry	38%	[150]
Vitamin C	n.s.	-	Dialysis followed by UV–Vis spectrophotometry	100%	[151]
Vitamin D	MLV	Triton X-100	Ultracentrifugation followed by HPLC	61.5%	[152]
Vitamin D_3_	n.s.	Methanol	Ultrafiltration in centrifuge tube followed by HPLC	74%	[127]
Vitamin D_3_	SUV/LUV	Methanol	HPLC	90.2%	[72]
Vitamin D_3_	SUV/MLV	Water	Ultrafiltration in centrifuge tube followed by RP-HPLC	100%	[153]
Vitamins D_3_ and K_2_	SUV/LUV	Ethanol	UV–Vis spectrophotometry	98%	[86]
Vitamin E	n.s.	Methanol	Dialysis followed by HPLC	92.5%	[154]
Vitamin E	n.s.	Sephadex® G25 M solution (10%, *w*/*v*) in double distilled water	Minicolumn centrifugation	98-101%	[155]
Vitamin E	n.s.	Chloroform/Methanol 2:1 *v*/*v*	UV–Vis spectrophotometry	78%	[156]
Vitamin E	n.s.	Water	Centrifugation followed by HPLC	94%	[157]
Vitamin E	n.s.	-	Centrifugation followed by UV–Vis spectrophotometry	83.8%	[158]
Vitamin E	n.s.	Chloride acid, Tween 80:ethanol and n-pentane	UV–Vis spectrophotometry	97%	[159]
Vitamin K_1_	n.s.	-	Ultracentrifugation followed by HPLC	79.2%	[160]
Vitamin K_1_	n.s.	Methanol	HPLC	3.4–154 µg/mg	[34]

n.s.: non specified.

## Data Availability

The data presented in this study are available on request from the corresponding author.

## Supplementary Materials

The following supporting information can be downloaded at: https://www.mdpi.com/article/10.3390/nano13091557/s1, 1. Post-processing methods that can be used during the production of vitamin-loaded liposomes (1.1 sonication, 1.2. extrusion, 1.3. high-pressure homogenization, 1.4 microfluidization, 1.5 dialysis, 1.6 freeze-thaw, and 1.7 lyophilization). References [193,194,195,196,197,198,199,200,201,202,203,204,205,206,207,208,209,210,211,212,213,214] are cited in the main text.
