# Supplementary material for: Current Applications of Liposomes for the Delivery of Vitamins: A Systematic Review
Source: Nanomaterials (Basel). 2023 May 5;13(9):1557. doi: 10.3390/nano13091557 (PMC10180326; doi:10.3390/nano13091557)
Supplement: Supplementary file 1 [file nanomaterials-13-01557-s001.zip › nanomaterials-2362452-supplementary.pdf]

nanomaterials

MDPI

Review

Current Applications of Liposomes for the Delivery of Bioactives: A Review on the Encapsulation of Vitamins

Matheus A. Chaves[1,2,†], Letícia S. Ferreira[1,†], Lucia Baldino[3,*], Samantha C. Pinho[1] and Ernesto Reverchon[3]

[1] Laboratory of Encapsulation and Functional Foods (LEnAlis), Department of Food Engineering, School of Animal Science and Food Engineering, University of São Paulo, Av. Duque de Caxias Norte, 225, Pirassununga, SP, Brazil; matheus.chaves@usp.br (M.A.C.); leticia2.ferreira@usp.br (L.S.F.); samantha@usp.br (S.C.P.)
[2] Laboratory of Molecular Morphophysiology and Development (LMMD), Department of Veterinary Medicine, School of Animal Science and Food Engineering, University of São Paulo, Av. Duque de Caxias Norte, 225, Pirassununga, SP, Brazil; matheus.chaves@usp.br (M.A.C.)
[3] Department of Industrial Engineering, University of Salerno, Via Giovanni Paolo II, 132, 84084, Fisciano, SA, Italy; ereverchon@unisa.it (E.R.)
† Authors contributed equally to this work.
* Correspondence: lbaldino@unisa.it; Tel.: +39089964356

Supplementary Material

1. Post-Processing Methods

1.1. Sonication

This process involves the use of high frequency sound waves to disrupt the liposome membrane and to reduce the size of liposomes. The sound waves create pressure waves in the liquid medium and lead the membranes to oscillate and eventually to rupture. Sonication can be performed using a variety of equipments, including ultrasonic baths, probe sonicators, and microtip sonicators. Ultrasonic baths are typically used for larger volumes; whereas probe and microtip sonicators are suitable for smaller volumes. Probes are preferred to form SUVs [193]. The main advantage of sonication is the relatively simplicity and requires only basic laboratory equipments. It can also be easily controlled by adjusting the duration and intensity of sonication, allowing for a precise control over the size and characteristics of the liposomes [194]. However, there are some potential drawbacks related to sonication, which include the high energy input when using probes that may cause thermal effects and consequently damages to heat-sensitive components, and the broad size distribution of liposomes, which may not be suitable for certain applications [53]. A study performed by Amiri et al. [195] developed a vitamin C-loaded nanoliposome by means of homogenization followed by sonication. Phospholipids, Chol, phytosterol powder and distilled water were used to produce the vesicles at increasing concentrations (0 - 100% w/w). After homogenization, different sonication times were tested to reduce the size of the vesicles (from 20 to 40 min) at a frequency of 23 kHz and amplitude of 12 μm, equipped with a 9.5 mm probe. The authors verified that from 20 to 25 min of sonication, increasing cholesterol to phytosterol ratio led to a decrease in vitamin C encapsulation, indicating a negative effect of the sonication time. On the other hand, at times higher than 35 min, the stability of vitamin C increased when a 25:25 Chol:phytosterols ratio was used. Negative effects were also perceived in the control release of vitamin C when increased sonication times were used. In Bochicchio et al. [144], vitamin B_{12}, vitamin D_2, and vitamin E-loaded LUVs were prepared by the thin film hydration method followed by sonication. Samples were prepared using PC, Chol, and L-α-phosphatidyl-DL-

glycerol sodium salt from egg yolk lecithin at a 10:8:1 molar ratio dissolved in chloroform/methanol 2:1 v/v. For vitamin D_2 and vitamin E-loaded vesicles, amounts of 14.92 mg and 3.70 mg of each vitamin were incorporated into the organic phase, respectively. After the evaporation of solvents, the lipid film was hydrated using PBS at pH 7.4. For vitamin B_{12}-loaded liposomes, 14.32 mg of vitamin were added to the hydration solution. Dispersions underwent through sonication at 45% amplitude, 20 Hz of frequency, and two ten-seconds rounds followed by twenty-second pause in order to prevent thermal disruption of the resulting LUVs. These authors observed that liposomes size ranged from 2.9 μm to 5.7 μm before sonication and from 40 nm to 51 nm after sonication. As expected, EE% of vitamins were all higher in the pre-sonicated samples (MLVs, EE% from 72% to 95%) than in vesicles post-sonication (LUVs, EE% from 56% to 76%).

1.2. Extrusion

This method involves passing the liposome dispersion through a series of membranes at defined pore sizes, which physically reduces the size of the liposomes. Different materials can be used throughout this process including polycarbonate, polyvinylidene difluoride, or cellulose acetate. The choice of the membrane depends on the specific application and the properties of the liposomes. Liposomes must be held above the gel to liquid crystalline transition temperature (T_m) to facilitate extrusion [196]. Manual extruders are available in the market; but only at small volumes (1 mL). One advantage of extrusion is that it is a gentle method that does not require high energy input or harsh conditions. This makes it suitable for downsizing liposomes containing sensitive components such as proteins and vitamins. However, extrusion can be more time-consuming and require more specialized equipment compared to other methods such as sonication. It is also important to optimize the conditions of pressure and temperature to achieve the desired size and characteristics of the liposomes [197].

Vitamin D_3-loaded liposomes were produced by Dalek et al. [153] by mixing propylene glycol containing 20% w/w of lipids and vitamin D_3, and purified water at a 1:1 w/w ratio followed by extrusion through 100 nm-sized polycarbonate membranes. These authors verified the possible precipitation of the vitamin before and after extrusion by means of HPLC. The results showed that no statistically significant losses occurred during the transformation of MLVs to SUVs during extrusion as vitamin retentions were up to 100% in both cases. Extrusion was also performed by Cansell, Moussaoui, and Lefrançois [138] to produce vitamin B_1-loaded micro-liposomes based on marine lipids and xanthan gum. The vesicles were first produced by hydrating a dry lipid film with a HEPES buffer containing the vitamin at 5 mg/mL. Polycarbonate filters with 5-μm pore size were used throughout a single extrusion and the loss of phospholipids was quantified by phosphorus assay. The authors found that xanthan gum allowed to triple the encapsulation efficiency of vitamin B_1 and stated that this behavior was possibly related to the interaction between superficial gum and vitamin dispersed in the external aqueous phase of liposomes.

1.3. High-Pressure Homogenization (HPH)

This method involves forcing the liposome dispersion through a narrow gap under high pressure, which creates shear forces that reduce the size of the liposomes. A main advantage of this method is its high reproducibility. Due to the fast acceleration of the liquid in the narrow gap, the hydrostatic pressure drops below the vapor pressure of the liquid and, therefore, steam bubbles are formed. The collapse of these cavitational bubbles is directly related to the mechanical energy that leads the liposomes to disrupt [193]. It can be more time-consuming than other downsizing methods and may require optimization of various parameters such as pressure, temperature, and number of passes to achieve the desired size and characteristics of liposomes. It requires up to a few

thousand psi to run and is generally more easily scalable to industrial processing than microfluidizers [197].

HPH was efficiently used by He et al. [198] to reduce the size of vitamin A-loaded cationic nanovesicles to ocular purposes. Several passes through the equipment (data not shown) resulted in vesicles with sizes up to 99 nm, zeta potential up to 53.7 mV and EE% up to 85%. In another study, Bi et al. [32] produced vitamin D_3-loaded nanoliposomes to act as anti-aging agents for the skin. These authors tested both the ethanol injection method followed by sonication for 5 min, and the hydration of a thin lipid film followed by sonication for 5 min and high-pressure homogenization (150 MPa). Samples that underwent through HPH were larger in size (55%) and encapsulated less (22%) vitamin than vesicles produced by the ethanol injection method followed by sonication. As aforementioned, several parameters have to be optimized when using HPH to obtain vesicles characterized by desired physicochemical parameters.

1.4. Microfluidization

This method involves forcing the liposome dispersion through a small orifice or channel under high pressure, which creates shear forces that reduce the size of the liposomes. Vesicles have to pass through the microfluidizer multiple times to obtain a uniform size distribution of smaller liposomes. Size reduction is caused by the collision of two fluid streams of dispersion at high-velocity in the interaction chamber [86]. The resulting liposomes show a narrow size distribution; but this method requires specialized equipments and is more expensive than other downsizing methods [12]. Differently from HPH apparatus, a microfluidizer typically requires higher pressures (up to tens of thousands of psi). As HPH, on the other hand, microfluidization is also time-consuming. This apparatus is suitable to treat small volumes of liposomes.

Yang et al. [200] produced vitamin C-nanoliposomes by film evaporation-dynamic high pressure microfluidization for skin purposes using SPC (1200 mg) and Chol (300 mg). Hydration was carried out using PBS at pH 6.8 and 150 mg of vitamin C and 600 mg of Tween 80. Samples were treated in a microfluidizer under 120 MPa for 2 passes. These authors verified that nanoliposomes presented better storage stability, sustained release and high skin penetration after microfluidization than before. This process was also adopted by Zhou et al. [201] to prepare vitamin C-loaded liposomes, containing different concentrations of high methoxyl and low methoxyl pectin. Samples were produced by the thin film hydration method using SPC (1000 mg), Chol (200 mg), and Tween 80 (300 mg). After the hydration using 90 mL of PBS at pH 6.8 and 300 mg of vitamin C, samples underwent microfluidization at 120 MPa pressure for two cycles. The authors verified that similar EE% were obtained regardless of the pectin coating and concentration (from 48.3% to 50.1%); but size and PDI increased when pectin concentration increased as well. After microfluidization, liposomal sizes ranged from 67 to 304 nm.

1.5. Dialysis

This method involves placing the liposome suspension in a bag or tube made of a semipermeable membrane and immersing it in a solution containing a lower concentration of salts or other solutes than the liposome dispersion. The membrane allows the smaller solutes and solvent molecules to pass through, while retaining the liposomes inside the bag or tube. As liposomes are dialyzed against the lower concentration solution, they lose water and salts to the surrounding solution, resulting in a reduction of their size [202]. The process can be controlled by varying the concentration gradient and time of dialysis to achieve the desired size and characteristics of the liposomes [203]. Advantages of dialysis include the gentleness of the method and the possibility to be used to remove impurities, unwanted components, or nonencapsulated material from liposome dispersions [204]. However, dialysis is a slow

process, and it may not be effective in reducing the size of liposomes beyond a certain limit. The size reduction achieved by dialysis may also be less uniform compared to other downsizing methods.

No literature regarding the use of dialysis to reduce the size of vitamin-loaded liposomes was found during this research. However, this method is currently used to verify the encapsulation efficiency of vitamins into liposomes by separating the free content from dispersions [145,150].

1.6. Freeze-Thaw

This method involves subjecting the liposome suspension to repeated cycles of freezing and thawing, which cause the liposome membrane to rupture and reform into smaller liposomes. MLVs are first frozen at a low temperature (usually -80 °C or lower) to cause the water inside the liposomes to expand and form ice crystals. The liposome suspension is then thawed at room temperature or in a water bath, allowing the ice crystals to melt and the liposomes to reform. The freeze-thaw cycles produce mechanical stress on the liposome membranes, leading to their disruption and the formation of smaller liposomes. The process can be repeated several times to achieve the desired size and characteristics of the liposomes [205]. One advantage of freeze-thawing is that it is a simple and low-cost method that does not require specialized equipment or expertise. Freeze-thawing can also be used to incorporate hydrophilic or hydrophobic molecules into the liposomes during the process. However, freeze-thawing can cause damage to the liposomes, leading to loss of encapsulated molecules and changes in the physical properties of the liposomes [206]. Cryoprotectant agents such as disaccharides, dimethyl sulphoxide and glycerol are commonly used to increase the resistance of phospholipid bilayers from damage during freeze-drying [207]. Therefore, this method may not be the best if a precise size and size distribution of vesicles are required.

Vitamin C-loaded liposomes were produced by Hudiyanti et al. [208] using the thin film hydration method followed by ultrasonication and hydration in a PBS buffer containing vitamin C. Phospholipids were extracted from sesame seeds. Freeze thawing sequences were performed for 5 min by cooling the samples at -5 °C, then heating at 50 °C, besides stirring. In these systems, EE% of vitamin C was up to 80%. Unden et al. [209] produced proteoliposomes encapsulating vitamin K_1. Mean diameters shown by the vesicles were about 100 nm. Battistelli, Salucci, and Falcieri [210] produced a multivitamin liposome by freezing a homogenous suspension of phosphoglycerides in water at -12 °C and, then, they were treated by a slow thawing at room temperature. The cycling was repeated and the liposomes were separated. Samples were then dried and resuspended in saline solutions. Vesicles obtained by this method were not homogeneous in diameter, as observed in TEM micrographs.

1.7. Freeze Drying / Lyophilization

Lipid-based nanoformulations of water-soluble drugs may experience leakage and drug degradation during preparation and storage. To address these issues, the freeze-drying method can be employed to remove water from the liposome systems when in a frozen state under low pressures [211]. As mentioned in the previous section, this step also involves the freezing of the aqueous solution containing the liposome formulation and subsequently the removing of ice by sublimation, which requires heat energy and low pressures. Initially, the product is frozen in a vial or flask at atmospheric pressure and then placed under a deep vacuum below the water triple point. Heating is then applied to cause the ice to sublime in a dry process (25 - 30 °C). After primary drying, a secondary drying under vacuum is required to desorb unfrozen water, followed by removing the dried product from the freeze dryer [212]. The freeze-drying method enables the production of stable liposome nanoformulations at preserved drug activity and minimizes production costs. The incorporation of cryoprotectants and

lyoprotectants into liposome formulations has been implemented to enhance product stability and functional properties after freezing and drying, respectively. To achieve optimal stability, the type and concentration of the saccharide must be optimized. While some theories have been proposed to explain the protective mechanisms of cryo/lyoprotectants during freeze-drying, further research is needed to fully understand their impact on liposome stability [212].

In this field, vitamin E-loaded liposomes were produced using PC at a 3:17 molar ratio vitamin:PC by hydration of a thin lipid film followed by extrusion. Lyophilization was applied to prolong the stability of the formulation during storage. Sucrose was added at a lipid/sucrose molar ratio of 1:5 before lyophilization. Final liposomes had a mean size of 130 - 140 nm and a PDI of 0.14. These authors stated that vitamin E content was preserved during storage of the lyophilized form at 2 - 8 °C for at least 6 months [213]. Liposomes containing vitamin A and ftorafur were prepared by Hua et al. [214] by atomization using soybean lecithin, Chol and poloxamer at a 1.2:0.6:0.4 mass ratio and hydrated with distilled water. Before lyophilization, different cryoprotectants were tested such as glucose, sucrose, mannitol, and trehalose. In this regard, a vitamin A EE% of 99% was obtained in freeze-dried samples when higher amounts of trehalose were added as cryoprotectants.
